# Biochemical and functional characterization of orf virus decapping protein OV71

**DOI:** 10.1186/s12985-025-03028-7

**Published:** 2025-11-27

**Authors:** Mandanda N. Mthethwa, Man-Lin Chang, Max R. Chang Ishcol, Ying-Fang Chen, Wei-Li Hsu, Guan-Ting Shen, Si-Yi Wu, Meng-Syun Li, Shin-Wu Liu

**Affiliations:** 1 Department of Veterinary Medicine, College of Veterinary Medicine, National Chung Hsing University, Taichung, Taiwan; 2 Graduate Institute of Microbiology and Public Health, College of Veterinary Medicine, National Chung Hsing University, Taichung, Taiwan

**Keywords:** Orf virus, OV71, Decapping enzyme, Nudix enzyme

## Abstract

**Background:**

Nudix enzymes constitute a family of hydrolases that share a conserved Nudix motif, which catalyzes the hydrolysis of **nu**cleoside **di**phosphates linked to another moiety **X**. Some members are cellular and viral decapping enzymes that hydrolyze the 5´ cap structure on an mRNA molecule. Unlike vaccinia virus, which encodes two Nudix enzymes, orf virus (ORFV) encodes only a single Nudix-containing gene, ORFV071 (OV71). This study investigates the biochemical properties of recombinant OV71 protein and its role in viral replication.

**Methods:**

In vitro decapping assays using radiolabeled capped RNA substrates were performed to assess OV71 activity in the presence or absence of competitors or metal cations. Electrophoretic mobility shift assays and pulldown assays evaluated the RNA-binding ability of OV71. Decapping-deficient mutant viruses were generated by homologous recombination, and their replication was analyzed using one-step growth curve experiments. Reverse transcription-qPCR quantified host and viral mRNA levels.

**Results:**

OV71 exhibited intrinsic decapping activity, hydrolyzing long capped RNAs to release m^7^GDP, with optimal activity in the presence of Mn^2+^. It bound both single- and double-stranded RNA and was expressed early during viral replication. Decapping-deficient mutant viruses replicated poorly in cells. Unlike the vaccinia virus decapping-deficient mutant, which triggers host antiviral responses leading to degradation of viral and host mRNAs as well as rRNAs, an orf virus mutant caused accumulation of host-capped RNAs and a severe reduction in viral mRNAs. Notably, host rRNA remained relatively intact compared to wild-type virus infection.

**Conclusion:**

OV71 is a decapping enzyme that hydrolyzes the cap structure on long capped mRNAs. It binds both single- and double-stranded RNA, suggesting that it may target both RNA species in infected cells. Its decapping activity is critical for efficient orf virus replication. Loss of this activity leads to the accumulation of host-capped mRNAs, a drastic reduction of viral mRNAs, and minimal impact on host rRNAs, indicating a role distinct from that of the vaccinia virus decapping enzymes.

**Supplementary Information:**

The online version contains supplementary material available at 10.1186/s12985-025-03028-7.

## Introduction

The regulation of mRNA degradation is a critical determinant of mRNA abundance and subsequent protein synthesis levels. In the major 5´ to 3´ eukaryotic mRNA decay pathway, degradation proceeds through coordinated sequential enzymatic actions including an initial shortening of the mRNA’s 3´ poly(A) tail by deadenylases, followed by removal of the 5´ cap by decapping enzymes, and finally the digestion of the RNA body by exonucleases. Each step involves cofactors that enhance the efficiency of all these enzymes [1]. This tightly regulated process is essential in various cellular and organismal processes, including cell proliferation and differentiation, immune response regulation, embryonic development, and aging [2–5]. Eukaryotic mRNAs possess a canonical 5´ cap structure consisting of a methylated guanosine linked to the first base by an unusual 5′ to 5′ triphosphate bridge (m^7^GpppN). This cap structure protects the mRNA from exonucleolytic attacks and facilitates cap-dependent translation in the cytoplasm [6–10]. Decapping enzymes, which remove this protective cap, are key regulators of mRNA turnover [11]. The most extensively studied eukaryotic decapping enzyme is Dcp2 [12–15], which contains a catalytic **nu**cleoside **di**phosphate linked to an **X** moiety (Nudix) hydrolase motif and is tightly regulated by multiple enhancers and an intrinsic auto-inhibitory mechanism [16–18].

Genes encoding Nudix proteins are found in diverse prokaryotic and eukaryotic organisms [19]. The Nudix proteins are hydrolases that act on a broad range of nucleoside diphosphate derivative substrates, such as (d)NTPs, nucleotide sugars, dinucleoside polyphosphates, coenzymes, ADP-ribose, and the 5′ cap structure of mRNA [20]. Nudix protein genes are also present in many large DNA viruses from multiple viral families within the phylum *Nucleocytoviricota*. The first identified viral Nudix decapping enzymes were D10 and D9 of vaccinia virus [21–24], the prototype member of the genus *Orthopoxvirus*. All known viruses that encode Nudix hydrolases possess an ortholog of the D10 gene, whereas the D9 ortholog is found only in most genera within the subfamily *Chordopoxvirinae*. In addition to D10 and D9, other characterized viral Nudix proteins include g5R from African swine fever virus (also referred to as African swine fever virus decapping enzyme (ASFV-DP) in another strain) [25, 26], and L375 from mimivirus [27]. These proteins function as mRNA decapping enzymes that hydrolyze the 5′ cap structure of mRNAs with sufficiently long RNA bodies, releasing m^7^GDP, in a manner mechanistically analogous to human decapping enzyme hDcp2 [21, 22, 25, 27, 28]. Treatment with excess uncapped RNA competitively inhibits cap hydrolysis, confirming that the RNA body is required for decapping activity [21, 22, 25, 27]. Methylated nucleotide analogs more effectively compete for decapping by D9, D10, and g5R than unmethylated ones [21, 22, 25, 29], and L375 fails to hydrolyze the unmethylated 5′ cap on mRNA [27], highlighting their specificity for methylated caps. The resolved co-crystal structure of D9 in complex with its decapping product, m^7^GDP, elucidates this preference: the methylated guanosine is sandwiched between two aromatic residues, F54 and Y158, through the π-π stacking interactions that specifically stabilize the methylated guanosine base over the unmethylated one [30].

The presence of viral decapping enzymes suggests complex interactions between RNA-mediated gene regulation and virus-host interactions. Transfected vaccinia virus D9 and D10 genes suppress translation of capped reporter RNAs [23]. Virally expressed D9 and D10, particularly D10, downregulate both host and viral mRNAs [24, 31, 32]. By removing caps from viral RNAs, D9 and D10 prevent the accumulation of viral double-stranded RNA (dsRNA), thereby avoiding activation of cellular dsRNA-mediated antiviral responses such as RNaseL-mediated RNA degradation and protein kinase R (PKR)-mediated translational arrest [33]. D10 preferentially targets host mRNAs transcribed from intron-containing genes, suggesting that splicing may be involved in regulating D10 activity [32]. D9 and D10 also appear to participate in pathways not directly related to decapping, as they synergize to promote selective translation of post-replicative viral transcripts containing a 5′ poly(A) leader sequence [34]. The African swine fever virus decapping enzyme g5R also exhibits potential multifunctionality. In addition to its decapping activity, it hydrolyzes diphosphoinositol polyphosphates (PP-InsPs), which are involved in vesicle biogenesis and trafficking, stress responses, and apoptosis [35–37], suggesting functions beyond mRNA decapping.

To date, D9 and D10 from the orthopoxvirus vaccinia virus are the only Nudix proteins in poxviruses to have been functionally characterized. In this study, we aimed to characterize Nudix protein ORFV071 (OV71) from the orf virus (ORFV), the prototype member of the genus *Parapoxvirus*. Orf virus, which possesses a smaller genome than vaccinia virus, is the causative agent of contagious pustular dermatitis. It is a zoonotic skin disease that primarily affects sheep and goats but can also infect humans [38, 39]. Infection typically produces localized, proliferative skin lesions, and the virus can repeatedly infect the same host, likely due to its diverse sets of immunomodulatory proteins that enable evasion of host innate immune responses [38, 39].

Unlike vaccinia virus, which encodes two decapping enzymes (D9 and D10), orf virus encodes only one, OV71. In this study, we cloned the OV71 gene, introduced point mutations to disrupt its catalytic activity, purified the recombinant wild-type and mutant OV71 proteins, and examined their biochemical properties. The biochemical functions of recombinant OV71 proteins are similar to other viral decapping enzymes: it exhibits intrinsic decapping activity, hydrolyzing the 5′ cap structure of mRNA but not the free cap, indicating a requirement of the RNA body for the decapping. OV71 also binds to both single- and double-stranded RNA, suggesting a role in modulating the levels of both RNA species in infected cells. Recombinant viruses were constructed to determine the role of OV71 in viral replication. Expression of OV71 was initiated early during viral infection. Deletion and catalytic mutants of OV71 appeared to be viable in cells, but exhibited severe growth defects, demonstrating that OV71 decapping activity is essential for optimal viral replication. Notably, OV71 promoted degradation of host-capped RNAs, which remained stable in cells infected with the OV71 catalytic mutant. In contrast, viral RNA accumulation was significantly delayed or nearly abolished in the mutant-infected cells, highlighting the critical role of OV71 decapping in maintaining viral gene expression. Notably, the host rRNA remained relatively unaffected. These findings differ from those of the vaccinia virus D9/D10 double decapping mutant, in which complete loss of viral decapping activity led to dsRNA accumulation and activation of host antiviral pathways that degraded not only host and viral mRNAs, but also host rRNA [33].

## Materials and methods

### *In Silico* analysis

The ORFV071 gene (OV71) in the orf virus Taiping strain was sequenced using Sanger sequencing, and the sequence was deposited in the GenBank database under the accession number OP820499. OV71 ortholog sequences from various viral and human species were retrieved from the NCBI database, with accession numbers listed in Table 1. Pairwise sequence comparison was performed using QIAGEN CLC Genomics Workbench 24.0.2 (QIAGEN). The alignment of the decapping enzyme Nudix motif amino acids was generated using Clustal Omega [40] and visualized by SnapGene Viewer version 8.0.2. The protein structure of orf virus OV71 and vaccinia D10 was predicted using the Colab AlphaFold 2.0 platform (https://colab.research.google.com/github/sokrypton/ColabFold/blob/main/AlphaFold2.ipynb) [41, 42] and visualized using the PyMOL Molecular Graphics System, version 3.1.1 Schrödinger, LLC. The surface charge of the AlphaFold2.0-predicted OV71 protein structure was analyzed using the Protein Sol Patches platform [43].

**Table 1:**
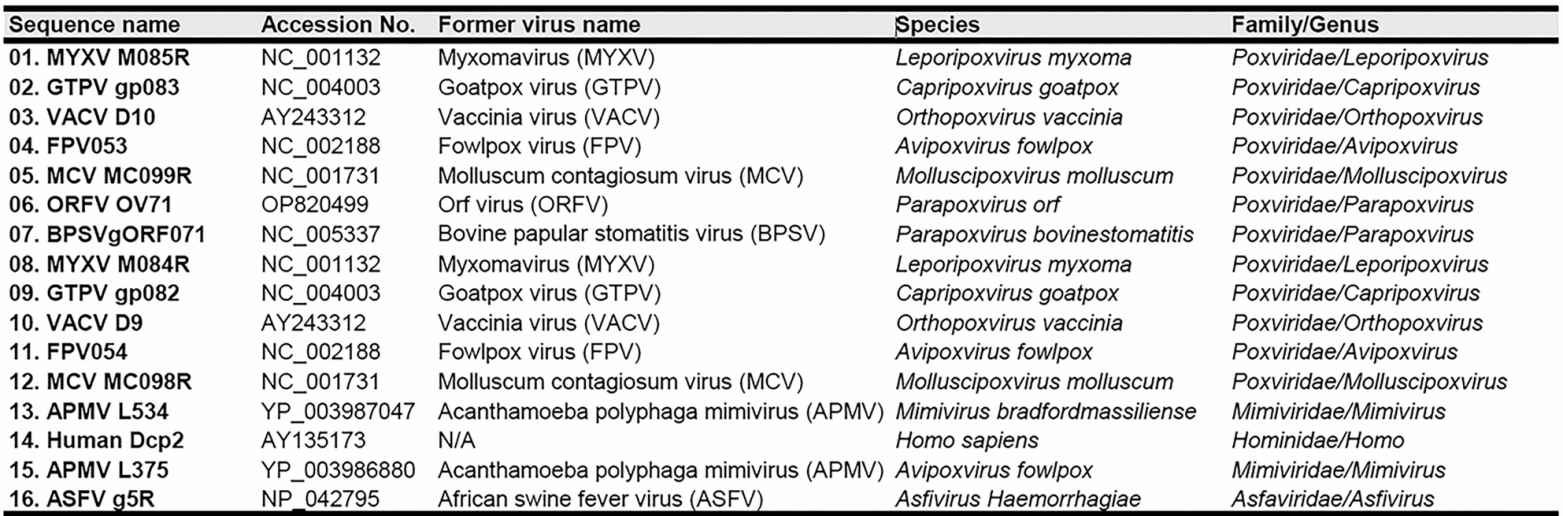
Detailed information of the reference sequence utilized for sequence comparison analysis

### Construction of expression plasmids

The ORFV071 gene (OV71) from the orf virus Taiping strain and the D9 gene from the vaccinia virus Western Reserve (WR) strain were amplified by PCR and cloned into the *Nde*I and *Xho*I restriction sites in the pET24a expression vector to generate the recombinant plasmids pET24-OV71 and pET24-D9, respectively. Site-directed mutagenesis was performed using overlapping PCR to introduce the catalytic site mutations E129Q/E130Q into the Nudix motif in the OV71 open reading frame, resulting in the mutant plasmid pET24-OV71mu. Additionally, OV71 and OV71mu were cloned into the *Bam*HI and *Xho*I sites in the pGEX-6p-1 plasmid, yielding pGEX-OV71 and pGEX-OV71mu, respectively. A truncated form of the orf virus OV20.0 gene, which includes a C-terminal deletion of amino acids 80–183 [44], was cloned into the *Nde*I/*Hin*dIII sites in pET24a to generate pET24-OV20ΔC.

### Expression and affinity purification of Recombinant proteins

Recombinant histidine-tagged OV71, OV71mu, and D9, used in the experiments shown in Figs. 2 and 3A, were purified by affinity chromatography using nickel-chelated Sepharose columns (GE Healthcare) following a modified manufacturer’s protocol. Briefly, the recombinant plasmids were used to transform competent BL21(DE3) *E. coli* cells. First, 0.5 L of the bacterial culture was grown and treated with 1 mM isopropyl-β-D-thiogalactopyranoside (IPTG) at 30 °C for 4 h to induce expression of recombinant proteins. The bacterial cells were then resuspended in a lysis buffer containing 50 mM Tris-HCl, 10 mM imidazole, 300 mM urea, and a commercial protease inhibitor (ProteaseArrest, Biosciences) at pH 7.4, followed by sonication to disrupt the cells. Then, the lysates were centrifuged at 12,000 g for 15 min to remove cell debris. Next, the clear supernatant containing the soluble recombinant protein was passed through a Sepharose column charged with 0.1 M NiSO_4_. The column was then washed three times using washing buffer 1 (50 mM Tris-HCl, 0.5 M NaCl, and 20 mM imidazole, pH 7.4) and another three times using washing buffer 2 (50 mM Tris-HCl, 0.5 M NaCl, and 50 mM imidazole, pH 7.4). The bound recombinant protein was sequentially eluted using elution buffer containing increasing concentrations of imidazole (50 mM Tris-HCl, 0.5 M NaCl, and 100–400 mM imidazole, pH 7.4), after which the eluted protein was dialyzed against phosphate buffered saline (PBS) (137 mM NaCl, 2.7 mM KCl, 10 mM Na_2_HPO_4_, and 1.8 mM KH_2_PO_4_, pH 7.4). The recombinant histidine-tagged OV71, OV71mu, and OV20ΔC proteins used in the experiments shown in Figs. 3B-D, 4 and 5 were purified using Talon cobalt-chelated resin columns (Clontech). The IPTG-induced bacterial cells were resuspended in Talon binding buffer (50 mM NaH_2_PO_4_, 300 mM NaCl, pH 7.0) followed by sonication. Next, the supernatant was passed through the column to allow the recombinant protein to bind. The column was subsequently washed four times with washing buffer (50 mM NaH_2_PO_4_, 300 mM NaCl, 5 mM imidazole, pH 7.0) and eluted with elution buffer (50 mM NaH_2_PO_4_, 300 mM NaCl, 150 mM imidazole, pH 7.0), after which the eluted protein was dialyzed against PBS or 20 mM Tris (pH 8.0). The glutathione S-transferase protein (GST) and GST-tagged OV71 and OV71mu proteins (GST-OV71 and GST-OV71mu) were purified using glutathione Sepharose 4B columns (Cytiva). BL21(DE3) cells transformed with pGEX-4T-1 (harboring a GST gene), pGEX-OV71, or pGEX-OV71mu were similarly induced by IPTG, resuspended in PBS, and sonicated. Each supernatant was passed through a glutathione Sepharose 4B column, washed six times with PBS, and eluted with elution buffer (10 mM reduced glutathione in 50 mM Tris-HCl, pH 8.0), after which each eluted protein was dialyzed against 20 mM Tris (pH 8.0).

### *In vitro* decapping assays

To generate the radiolabeled capped RNA probe for decapping assays, a 206-nucleotide (nt) uncapped cold RNA molecule was in vitro transcribed using SP6 RNA polymerase (Promega) and a PCR-generated DNA template containing the SP6 promoter. The DNA template was generated by amplifying the multiple cloning site region of the pcDNA3.1 plasmid with the introduced SP6 promoter sequence at the 5’ end. After in vitro transcription, the unincorporated NTPs (ATP, UTP, GTP, CTP) were removed using a MicroSpin gel filtration column (Bio-Rad). The RNA was capped using the vaccinia virus capping enzyme (New England Biolabs) in the presence of 1 µM [α-^32^P]GTP radioisotope and 1 mM S-adenosyl methionine (SAM) to generate the resultant cap-labeled RNA, in which the phosphate linked to the 7-methylguanosine was radiolabeled. The labeled cap structure without the RNA body was generated by digesting the cap-labeled RNA with 1 unit nuclease P1 (New England Biolabs) at 37 °C for 1 h. In each in vitro decapping assay, 4 or 8 pmol of each indicated recombinant protein was incubated with 0.05 pmol cap-labeled RNA in the standard decapping buffer (100 mM potassium acetate, 10 mM Tris-HCl, 2 mM MgCl_2_, 0.5 mM MnCl_2_, 2 mM DTT, pH 7.5) [21] at 37 °C for 15 min. The decapping products were spotted onto polyethyleneimine (PEI) cellulose plates (Sigma-Aldrich) and resolved by thin-layer chromatography (TLC) using 0.45 M (NH_4_)_2_SO_4_ as the mobile phase. Plates were then air-dried and exposed to X-ray films (FUJIFILM).

### Electrophoretic mobility shift assay (EMSA)

The uniformly internally labeled 206-nt uncapped RNA was generated by in vitro transcription in the presence of [α-^32^P]UTP using the same DNA template used to generate the capped RNA in the decapping assays. The generated RNA probe was electrophoresed in a denaturing 6% polyacrylamide gel containing 7 M urea. Then, the gel was exposed to an X-ray film (FUJIFILM) to identify the major probe band, which was then excised and ground with a pipetman tip. Subsequently, the ground gel pieces were resuspended in the elution buffer (0.5 M ammonium acetate trihydrate, 1 mM EDTA, 0.1% SDS, pH 8.0) and incubated at 37 °C overnight. Next, the supernatant was cleaned using chloroform, and the RNA probe was precipitated by isopropanol. The EMSA was performed using a protocol modified from Tsai et al. [45]. Briefly, the indicated protein was incubated with 0.05 pmol RNA probe in the binding buffer (20 mM HEPES, 10 mM EDTA, 1 M KCl, 1 mM DTT, 80 µg/mL BSA, 50% v/v glycerol) on ice for 15 min and resolved on a 6% native TBE (tris-borate-EDTA) acrylamide gel. Finally, the gel was dried and exposed to an X-ray film (FUJIFILM).

### ssRNA and polyi: C pulldown assays

For ssRNA pulldown assays, an internally biotinylated RNA substrate was generated by in vitro transcription using the same DNA template for the decapping assays. The reaction contained a mixture of regular NTPs and biotinylated UTP (Roche) to enable internal biotin incorporation. The generated biotinylated RNA was extracted using the RNeasy^®^ Mini kit (QIAGEN) following the manufacturer’s instructions. For the pulldown assays, 1.5 µg of purified GST-tagged proteins or 0.5 µg of histidine-tagged proteins were incubated with 1.5 µg of in vitro transcribed internally biotinylated RNA or 0.1 µg biotinylated polyI: C (InvivoGen) in regular decapping buffer (100 mM potassium acetate, 10 mM Tris-HCl, 2 mM MgCl_2_, 0.5 mM MnCl_2_, 2 mM DTT, pH 7.5) at room temperature for 1 h. After incubation, 10% of the reaction volume was separated and stored in a freezer at -20 °C. The remaining reaction was mixed with washed streptavidin beads and incubated at room temperature for 1 h. The streptavidin beads were then washed and resuspended in SDS-PAGE loading buffer and heated at 95 °C for 5 min. The supernatant containing the eluted protein (E) was separated from the beads (B), and 10% of the reaction that had been previously separated was used as the input (I). The input (I), eluted fractions (E), and beads (B) were resolved on an SDS-PAGE gel and analyzed by Western blotting.

### Cell maintenance and virus propagation

HEK293T cells and primary goat fibroblast (FB) cells were cultured in Dulbecco’s Modified Eagle Medium (DMEM) supplemented with 100 units/mL penicillin, 100 µg/mL streptomycin (Cytiva), 0.5 µg/mL amphotericin B (Gibco), and 10% fetal bovine serum (FBS) (Avantor Seradigm). All of the orf viruses were propagated and titered using FB cells.

### Construction of recombinant viruses

For the construction of the recombinant virus vOV71flag, a DNA fragment containing an approximate 500 bp OV71 3´ region with a 3xFlag tag sequence at the end, followed by an EGFP (enhanced green fluorescent protein) open reading frame driven by an upstream modified vaccinia virus H5 promoter [46, 47], and a 500 bp 5´ partial region of the OV72 sequence was cloned into a pcDNA3.1 vector to form the transfer plasmid. To perform homologous recombination, wild-type (WT) orf virus was used to infect 5 × 10^5^ HEK293T cells at an MOI of 0.1 for 2 h, followed by transfection of 4 µg transfer plasmid using Lipofectamine 2000 (Thermo Fisher). After 24 h, the cells were lysed and diluted and used to infect fresh FB cells. The plaques emitting green fluorescence were selected and used to infect another set of fresh FB cells, and the selection was performed for five cycles. vΔOV71 was constructed using a similar methodology, while the OV71 open reading frame was replaced by the H5 promoter-driven EGFP gene, followed by seven selection cycles. To construct the revertant virus vRev-WT, a transfer plasmid was generated by inserting a DNA fragment containing the wild-type OV71 open reading frame with adjacent partial sequences of the 3´ region of OV70 and the 5´ region of OV72, into the *Hin*dIII and *Not*I restriction sites of the pcDNA3.1. HEK293T cells were infected with vΔOV71 at an MOI of 2, followed by transfection of the transfer plasmid. The colorless viral plaques were selected for four cycles. To construct the catalytic mutant virus vOV71mu, a fragment containing the flanking OV70 3´ region, the entire OV71 open reading frame with E129Q/E130Q mutations at the catalytic domain, and the flanking OV72 5´ region was generated by overlapping PCR and cloned into pcDNA3.1 to form the resultant transfer vector. During the site-directed mutagenesis procedure, a *Bam*HI site was introduced immediately before the Q129 residue without disrupting the OV71 open reading frame. The rest of the homologous recombination and selection procedure was similar to that used to construct vRev-WT.

### Western blot analysis

Proteins were electrophoresed on 12% or 15% SDS-polyacrylamide gels and electrotransferred to a PVDF membrane using a mini-blot module (Invitrogen). Then, the membranes were blocked using 5% nonfat milk in PBS for 1 h, washed with PBST (0.05% Tween-20 in PBS), and incubated with the primary antibody at 4 °C overnight. Next, the membrane was washed with PBST and incubated with the secondary antibody conjugated with the horseradish peroxidase (HRP) at room temperature for 1 h. The antibodies were diluted in 5% nonfat milk in PBS with the following dilution ratios: anti-GST antibody (1:5000; Yao-Hong Biotechnology), anti-histidine antibody (1:20000; Bio-Rad), M2 anti-Flag antibody (1:5000; Sigma-Aldrich), anti-β-actin antibody (1:5000; Novus Biologicals), and goat anti-mouse secondary antibody (1:5000; Jackson ImmunoResearch). After incubation with the secondary antibody, the membrane was washed with PBST, and the bound target proteins were visualized by adding the SuperSignal West Pico PLUS chemiluminescent substrate (Thermo Scientific), followed by exposure to an X-ray film (FUJIFILM) or detection by a Multigel-21 image system (TOPBIO, Taiwan).

### One-step virus growth and plaque assay

Primary goat FB cells were infected with viruses at an MOI of 1. After 2 h of adsorption, cells were washed three times with PBS and covered with infection media containing DMEM supplemented with 2.5% FBS and antibiotics. At different time points, cells were harvested by scraping or trypsinization and then subjected to three freeze-thaw cycles for cell lysis to release viral particles. Lysates were then serially diluted and used to infect fresh FB cells. After 2 h of absorption, the supernatant was replaced with DMEM containing 2.5% fetal bovine serum (FBS), antibiotics, and 0.5% methylcellulose. The cells were incubated at 37 °C for 10–15 days to allow plaque formation. Plaques were then fixed and stained with 0.1% crystal violet in 75% ethanol or immunostained using a homemade anti-F1L antibody [48] (described below) for visualization, and viral titers were subsequently determined.

### Immunocytochemistry (ICC)

Viral plaques were immunostained using a homemade anti-F1L antibody generated in rabbits [48], which recognizes the F1L membrane protein of orf virus. The immunostaining procedure was performed with the Dako REAL EnVision Detection System (Agilent Technologies Singapore, Singapore) according to the manufacturer’s instructions. Briefly, infected cells were fixed with 80% acetone and incubated with the anti-F1L antibody at a 1:500 dilution. After rinsing with PBS, the cells were treated with the HRP-conjugated secondary antibody provided in the kit, and color was developed using the DAB substrate solution.

### Quantitative PCR (qPCR) and reverse transcription-quantitative PCR (RT-qPCR)

The relative viral genome levels were determined by quantitative PCR (qPCR). A set of goat fibroblast (FB) cells was infected with vOV71flag at an MOI of 12. After viral adsorption for 2 h, the mock-infected and virus-infected cells were washed three times with PBS and then incubated with fresh infection media containing 2.5% FBS and antibiotics. Cells were harvested at 2, 6, 10, and 22 h postinfection. In a separate group of cells, 50 µg/ml cytosine arabinoside (AraC), a DNA replication inhibitor, was added 1 h prior to viral infection. AraC was maintained in the infection media throughout the entire course of infection. Total DNA of each sample was extracted using QIAamp DNA Kit (QIAGEN). One-twelfth of the total DNA (~ 90 ng) was subjected to qPCR using OV71-specific primers (OV71 qPCR for/OV71 qPCR rev in the Supplementary Excel file). Standard curves were generated using serial dilutions of the plasmid pET24-OV71 to quantify absolute viral genome copy numbers.

In parallel sets of experiments, cellular or viral transcript levels were determined by reverse transcription-quantitative PCR (RT-qPCR). The total RNA was isolated using the RNeasy Mini kit (QIAGEN). One microgram of total RNA was reverse transcribed into cDNA using the HiScript III RT SuperMix for qPCR kit (Vazyme). Quantitative PCR (qPCR) was carried out using a 2x MorreSYBR qPCR Master Mix reagent (MORREBIO) with oligos corresponding to designated genes in a LightCycler^®^ 96 Instrument (Roche). The sequences of the used primers are listed in the Supplementary Excel file. The PCR conditions were 95 °C for 10 s, followed by 40 cycles of 95 °C for 10 s and 60 °C for 30 s. The relative RNA level of each gene was calculated using the 2^−ΔΔCT^ method [49], using 18 S rRNA as the internal reference gene.

### Electrophoresis of cellular total RNA on Glyoxal denaturing gels

Total RNA extracted from mock-infected or virus-infected cells, as described in the previous RT-qPCR section, was resolved on a 1% denaturing agarose gel prepared with the NorthernMax™ Glyoxal Gel Buffer (Invitrogen).

### Statistical analysis

The data were plotted using GraphPad Prism, version 9.4.1. The data are presented as the mean ± SEM. Student’s t-tests were conducted to assess the statistical significance of the differences between sample groups, with *, **, ***, and **** indicating *P* < 0.05, *P* < 0.01, *P* < 0.001, and *P* < 0.0001, respectively.

## Results

### Orf virus genome encodes a putative protein ORFV071 (OV71) that contains a nudix motif

**Fig. 1:**
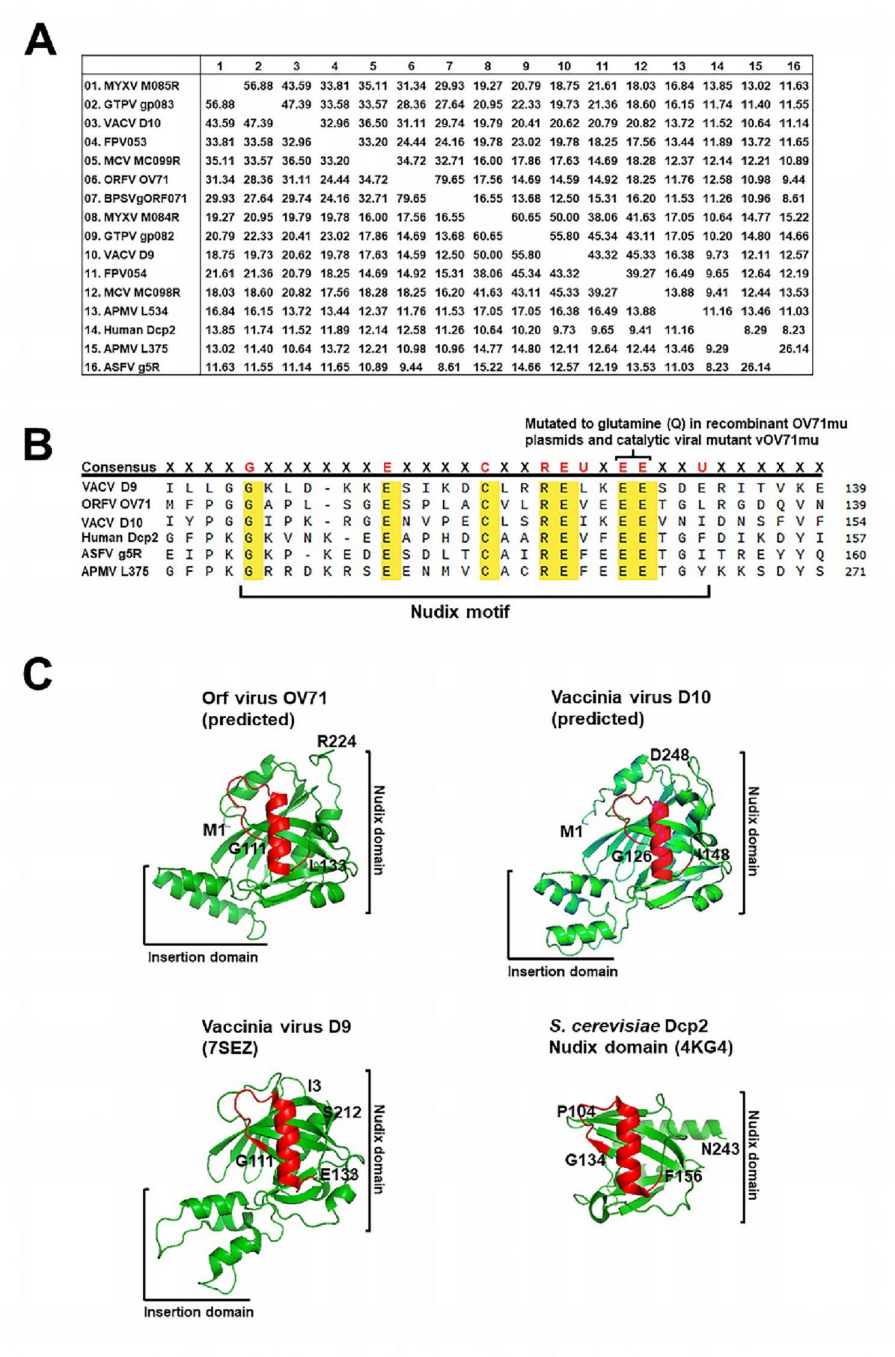
Comparative analysis of amino acid sequences and structures of viral and yeast Nudix proteins. (**A**) Pairwise amino acid sequence identity matrix comparing 15 D10 and D9 orthologs from nucleocytoplasmic large DNA viruses and the human Dcp2 decapping enzyme. Percentages indicate sequence identity. The matrix was generated using QIAGEN CLC Genomics Workbench 24.0.2 (QIAGEN). Accession numbers for the reference sequences are listed in Table 1. (**B**) Amino acid sequence alignment of the Nudix motif from five viral decapping enzymes and the human Dcp2 decapping enzyme. The alignment was performed using Clustal Omega. The two adjacent glutamic acid residues previously shown to be essential for catalytic activity are highlighted. In this study, these residues were substituted with glutamines (Q) for subsequent biochemical and viral replication analyses. (**C**) Predicted structures of OV71 and vaccinia virus D10, along with the experimentally resolved structures of vaccinia virus D9 (PDB accession code 7SEZ) [30], and the Nudix domain of *Saccharomyces cerevisiae* decapping enzyme Dcp2 (PDB accession code 4KG4) [53]. The predicted structures of OV71 and D10 were generated using Colab AlphaFold 2.0 and visualized with the PyMOL Molecular Graphics System. The loop-helix-loop regions corresponding to the Nudix motif are highlighted in red. In OV71 and D10, all residues within the highlighted Nudix motif have pLDDT (predicted local distance difference test) scores above 85. For each structure, the terminal residues of the Nudix motif and the full-length protein are labeled

Over the past two decades, the identification of novel poxviruses has expanded the *Chordopoxvirinae* subfamily to include 18 genera as of 2023 [50]. Among them, only the *Parapoxvirus* and *Salmonpoxvirus* encode a single Nudix hydrolase ortholog corresponding to vaccinia virus D10R (D10; VACVWR_115). In contrast, viral genomes from the remaining 16 genera contain two adjacent open reading frames corresponding to vaccinia virus D9R (D9; VACVWR_114) and D10R. Figure 1A presents a matrix of amino acid sequence identity between orf virus OV71 and 14 other viral Nudix proteins, including D9 and D10 orthologs from *Chordopoxvirinae* members, L375 and L534 from Acanthamoeba polyphaga mimivirus (*Mimiviridae*), g5R from African swine fever virus (*Asfaviridae*), and the human decapping enzyme Dcp2 (hDcp2). Detailed information of the selected sequences, based on the most recent updates from the International Committee on Taxonomy of Viruses (ICTV), is summarized in Table 1.

OV71 shares the highest sequence identity with BPSVgORF071, the D10 ortholog in bovine papular stomatitis virus (BPSV), also belonging to the *Parapoxvirus* genus. Additionally, OV71 shares over 30% identity with D10 orthologs from several viruses in other genera, including MC099R (34.72%) from molluscum contagiosum virus (MCV), M085R (31.34%) from myxomavirus (MYXV), and D10 (31.11%) from vaccinia virus (VACV), in agreement with previous phylogenetic and comparative genomic analyses [51]. In contrast, OV71 shares low sequence identity with more distantly related viral decapping enzymes, such as 11.76% with L534 and 10.98% with L375 from Acanthamoeba polyphaga mimivirus (APMV).

Consistent with other D10 orthologs, OV71 contains a conserved Nudix motif. As shown in Fig. 1B, sequence alignment of the OV71 Nudix motif with four other viral decapping enzymes and human Dcp2 reveals that OV71 Nudix motif shows perfect homology to the consensus Nudix motif sequence GX_5_EX_7_REUXEEXGU (where U represents isoleucine, leucine, or valine, and X represents any amino acid). Previous studies have demonstrated that the two adjacent glutamic acid residues (E-E, highlighted in Fig. 1B) are essential for the catalytic activity, as mutation of these residues completely abolishes decapping function [12, 21, 22, 25]. In this study, the corresponding residues E129 and E130 in OV71 were also mutated to assess their roles in catalysis and viral replication in infected cells. Figure 1C shows the predicted structure of OV71 generated by AlphaFold 2.0 [41, 42]. Similar to the experimentally resolved structures of vaccinia virus D9 and the Nudix domain of yeast Dcp2, as well as the AlphaFold2.0-predicted structure of D10, OV71 adopts a typical α-β-α Nudix fold. This Nudix fold consists of two mixed β-sheets sandwiched between two α-helices, one of which includes the 23-residue Nudix helix. The Nudix motif itself forms a conserved loop-helix-loop structure, consistent with other Nudix family proteins [52]. In the D9 structure, an insertion domain containing a bundle of three helices provides an important residue (F54) to interact with the bound m^7^GDP [30]. A similar insertion domain is present in the predicted structures of OV71 and D10 (Fig. 1C). The structural similarity of OV71 to other viral decapping enzymes suggests that OV71 may retain conserved enzymatic activity.

### OV71 hydrolyzes the 5´ cap in capped RNA to release m^7^GDP

**Fig. 2:**
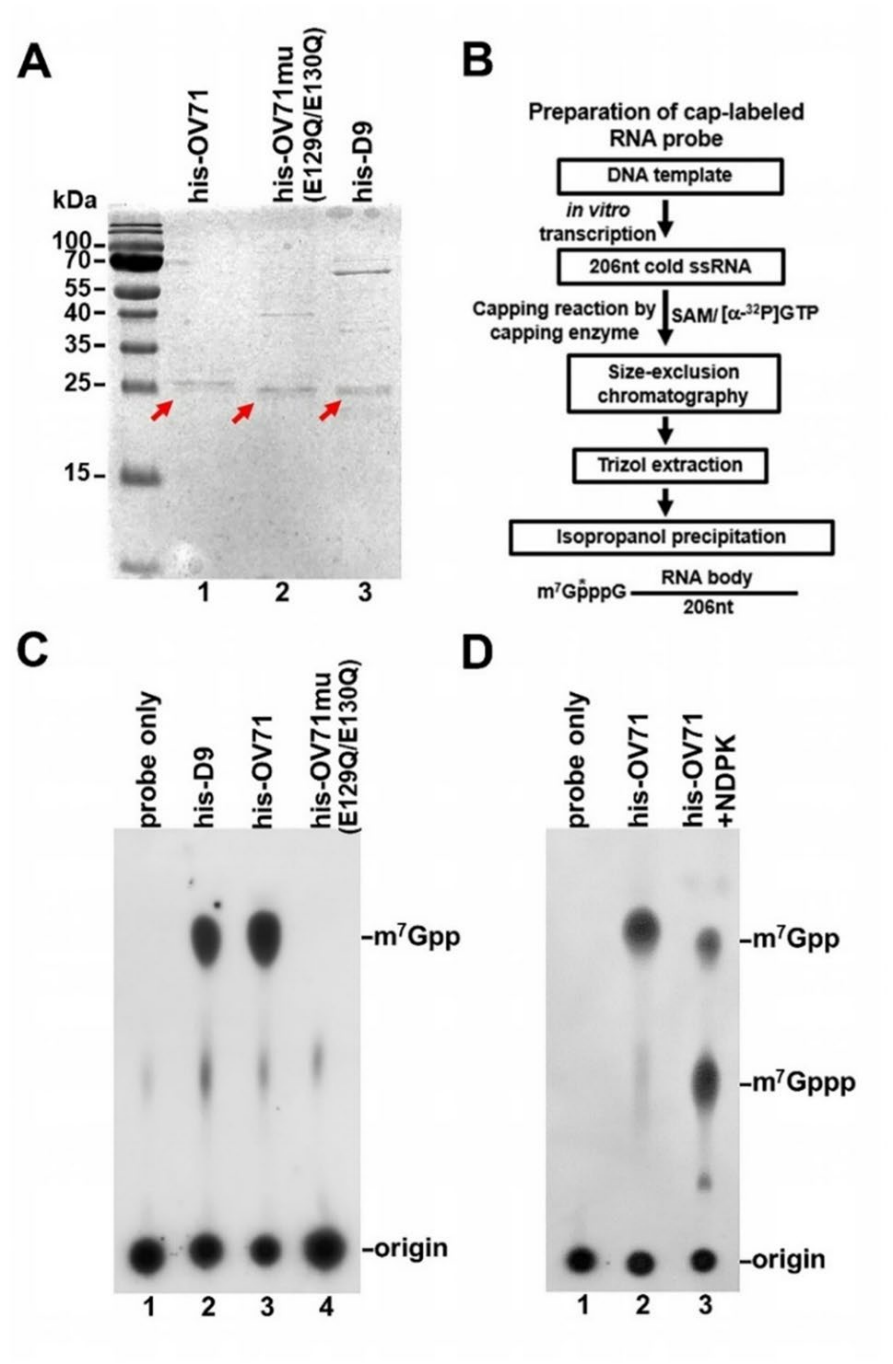
OV71 hydrolyzes the 5´ cap in capped RNA to release m^7^GDP. (**A**) SDS-PAGE analysis of purified recombinant proteins. Histidine-tagged wild-type OV71 (his-OV71), the catalytic mutant OV71mu (his-OV71mu) harboring the E129Q/E130Q substitutions, and vaccinia virus D9 (his-D9) were expressed in *E. coli* and purified by nickel-affinity chromatography using nickel-chelated Sepharose columns (GE Healthcare). Proteins were resolved on a 12% SDS-PAGE gel and stained with Coomassie Blue. The bands corresponding to purified decapping proteins are indicated by small arrows. These purified proteins were used in the decapping assays shown in this figure and Fig. 3A. (**B**) Schematic of the procedure used to generate the 206 nt radiolabeled capped RNA substrate. In vitro transcription was performed using a DNA template containing an SP6 promoter to generate a cold 206 nt RNA. The RNA was capped using the vaccinia capping enzyme in the presence of S-adenosyl methionine (SAM) and [α-^32^P]GTP. The capped RNA was purified by size-exclusion chromatography to remove unincorporated nucleotides, followed by TRIzol™ (Invitrogen) extraction and isopropanol precipitation. (**C**) Decapping assay using 8 pmol of each recombinant protein incubated with approximately 0.05 pmol of cap-labeled RNA in regular decapping buffer (100 mM potassium acetate, 10 mM Tris-HCl, 2 mM MgCl_2_, 0.5 mM MnCl_2_, 2 mM DTT, pH 7.5). Reaction products were resolved on polyethyleneimine (PEI) cellulose thin-layer chromatography (TLC) plates using 0.45 M (NH_4_)_2_SO_4_ as the mobile phase. (**D**) Nucleoside diphosphate kinase (NDPK) converted m^7^GDP to m^7^GTP. Eight picomoles of recombinant his-tagged OV71 were used, as in (**C**). In lane 3, the reaction products were incubated with NDPK in the presence of 1 mM ATP. The resulting product co-migrated with cold commercial m^7^GTP, as detected by UV shadowing, confirming that the original decapping product was m^7^GDP

The OV71 open reading frame was cloned into the prokaryotic expression vector pET24a to evaluate its intrinsic decapping activity. To test the importance of the conserved glutamic acid residues E129 and E130 within the Nudix motif, corresponding to the essential Nudix glutamic acids in other decapping enzymes, both residues were mutated to glutamine (Q), generating a mutant gene OV71mu, which is expected to encode a catalytically inactive form of OV71. OV71mu was cloned into pET24a similarly to the wild-type gene. As a positive control, the open reading frame of the vaccinia virus decapping enzyme D9 was also cloned. His-tagged versions of all three proteins (OV71, OV71mu, and D9) were expressed in *E. coli* and purified by nickel-affinity chromatography, followed by SDS-PAGE analysis. Each sample showed a predominant band at approximately 25 kDa, consistent with the expected size of the recombinant proteins (Fig. 2A). A 206 nt radiolabeled-capped RNA substrate was synthesized as described in Materials and Methods (Fig. 2B). Incubation of the capped RNA with OV71 produced a decapping product that migrated to the same position as the m^7^GDP generated by D9 (Fig. 2C, lanes 2 and 3). In contrast, the catalytically inactive OV71mu failed to produce this decapping product (Fig. 2C, lane 4), confirming that E129 and E130 are essential for OV71 decapping activity. To verify the identity of the decapping product as m^7^GDP, the OV71 decapping reaction was treated with nucleoside diphosphate kinase (NDPK), which phosphorylates nucleoside diphosphates to form nucleoside triphosphates [12]. As expected, the majority of the decapping product was converted to a slower-migrating product that co-migrated with the commercial cold m^7^GTP, as detected by UV shadowing (Fig. 2D, lane 3), confirming that the original decapping product was m^7^GDP.

### The RNA moiety of capped RNA is essential for OV71 decapping activity

**Fig. 3:**
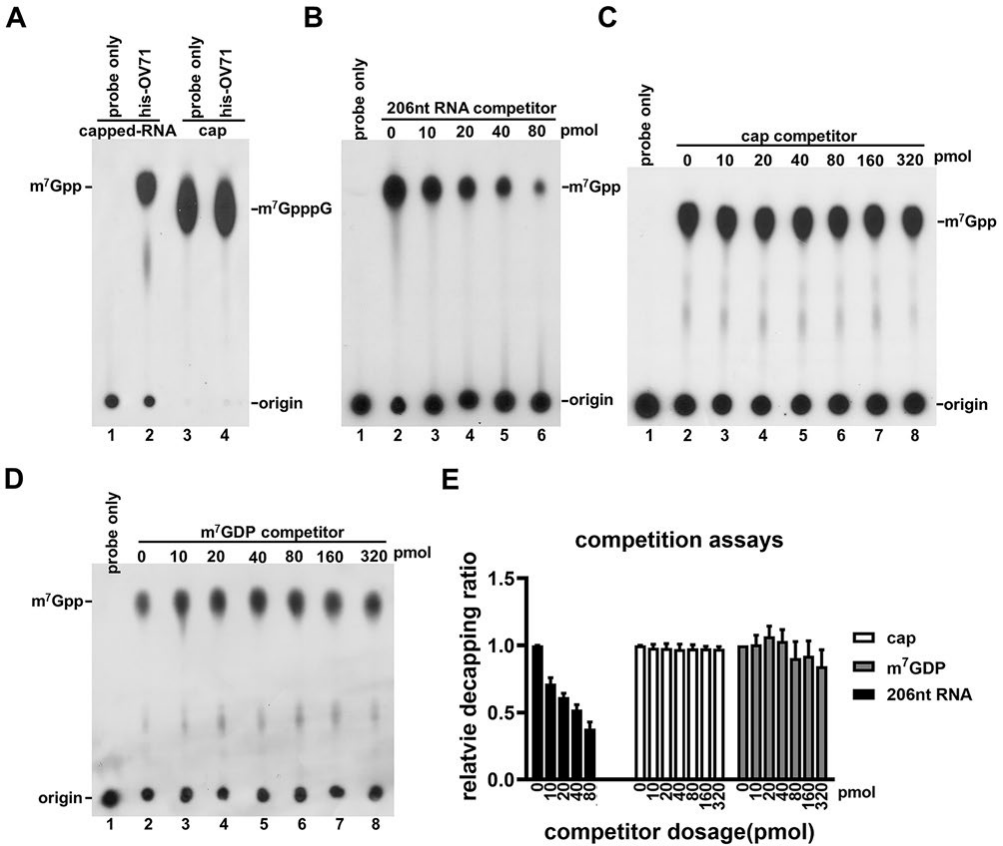
RNA body in capped RNA is required for OV71 decapping activity. (**A**) Radiolabeled-capped RNA and the cap structure lacking the RNA body were used as substrates in decapping assays with recombinant his-OV71. The cap structure (m^7^GpppG) was generated by digesting the RNA body from the capped RNA using nuclease P1. (**B-D**) Increasing amounts of 206 nt uncapped RNA, cap structure (m^7^GpppG), and the decapping product m^7^GDP were tested as competitors in his-OV71 decapping assays. (**E**) The percentage of decapping was quantified by measuring the exposed area of m^7^GDP and dividing it by the sum of the areas of m^7^GDP and the origin. The areas of m^7^GDP and spotted origins were measured using ImageJ1.53 K (https://imagej.net/ij/). Relative decapping ratios were normalized to reactions containing no competitor. The relative decapping ratios were plotted against competitor amounts using GraphPad Prism 10.4.1. The error bars represent the standard error of the mean (SEM)

Several Nudix decapping enzymes preferentially act on the capped RNA rather than the cap structure lacking an RNA body [21, 22, 28, 54]. In contrast, the eukaryotic scavenger decapping enzyme DcpS, which lacks a Nudix motif, hydrolyzes residual cap structures without the RNA body in the 3´-5´ mRNA degradation pathway [55–57]. Having demonstrated OV71’s ability to decap intact capped RNA, we next tested whether OV71 could also hydrolyze the free cap structure in the absence of its RNA moiety. The capped RNA prepared for the experiments in Fig. 2 was treated with nuclease P1 to digest the RNA body, leaving the cap structure m^7^GpppG. This product was then subjected to a decapping assay with OV71. No hydrolysis of the cap was observed (Fig. 3A, lanes 3 and 4), indicating that OV71 does not act efficiently on the cap alone and, like other Nudix decapping enzymes, requires the RNA moiety for substrate recognition and catalytic activity.

### Uncapped RNA inhibits OV71 decapping activity

Given that the RNA body of capped RNA is essential for OV71 decapping, we hypothesized that OV71 recognizes and binds the RNA moiety to access the cap structure, similar to human Dcp2 and vaccinia virus D9 and D10. If true, excess uncapped RNA should competitively inhibit decapping by sequestering OV71. To test this hypothesis, increasing concentrations of uncapped 206 nt RNA were added to decapping reactions containing 4 pmol of OV71. As shown in Fig. 3B, the presence of uncapped RNA inhibited decapping activity. Specifically, 40 pmol of uncapped RNA reduced decapping by approximately 50% (Fig. 3E), supporting the hypothesis that OV71 binds the RNA body to engage the cap structure. In contrast, even an 80-fold molar excess (320 pmol) of the cap analog (m^7^GpppG) had no inhibitory effect on decapping (Fig. 3C and E), suggesting that OV71 has minimal affinity for the cap structure without the RNA body. A higher concentration (> 80 pmol) of the decapping product m^7^GDP modestly inhibited the decapping (Fig. 3D and E), indicating weak affinity between OV71 and its decapping product m^7^GDP. Collectively, these results support a model in which OV71 accesses the cap structure via interactions with the RNA body of the capped RNA substrate.

### OV71 has an affinity for both single- and double-stranded RNA

**Fig. 4:**
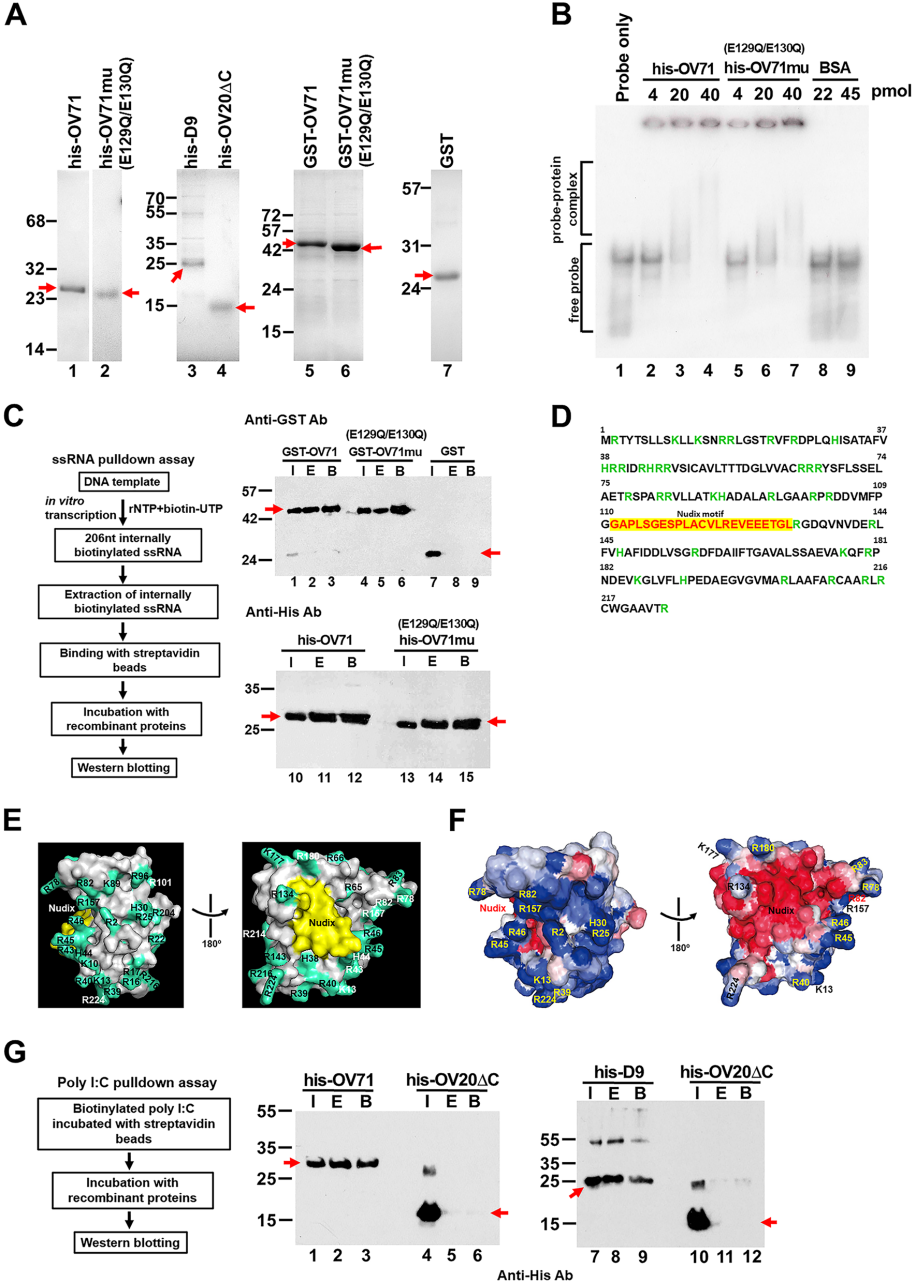
OV71 has an affinity for both single-stranded RNA (ssRNA) and double-stranded RNA (dsRNA). (**A**) Purified his-and GST-tagged recombinant proteins were resolved on 12% or 15% SDS-PAGE gels and visualized by Coomassie blue staining. The his-OV71mu and GST-OV71mu proteins harbor the catalytic E129Q/E130Q mutations within the OV71 open reading frame, which abolish the decapping activity as shown in Fig. 2C (lane 4). The his-OV20ΔC protein is a C-terminal truncation mutant of OV20.0 lacking dsRNA-binding ability and was used as a negative control [44]. His-tagged proteins were purified using cobalt-chelated columns (Clontech), and GST-tagged proteins were purified using glutathione Sepharose 4B columns (Cytiva). The bands corresponding to purified his-and GST-tagged decapping proteins are indicated by small arrows. (**B**) Electrophoretic mobility shift assay (EMSA). A 206 nt gel-purified internally radiolabeled RNA probe was incubated with the indicated proteins and resolved on a 6% native polyacrylamide gel. (**C**) Pulldown assays using a 206 nt internally biotinylated ssRNA bound to streptavidin beads, followed by incubation with the indicated proteins. Eluted proteins (**E**) were analyzed by Western blotting with anti-GST (upper panel) or anti-His (lower panel) antibodies. GST protein served as the negative control, as it did not bind RNA in another study [25]. **I**: input (10% of binding reaction); **E**: eluted fractions from the beads; **B**: beads. (**D**) OV71 amino acid sequence, with basic residues arginine (R), lysine (K), and histidine (H) highlighted in green and the Nudix motif highlighted in red. (**E**) The surface structure of AlphaFold2.0-predicted OV71 displayed by PyMOL Molecular Graphics System (version 3.1.1). The Nudix motif is highlighted in yellow; basic residues are shown in cyan-green. (**F**) The surface charge of the structures shown in (**E**) was analyzed by Protein Sol Patches platform [43]. Positively charged regions are shown in blue and negatively charged regions in red; the scale of charge distribution ranges from − 75mV to + 75mV. (**G**) Pulldown assays with biotinylated polyI: C (a dsRNA analog) to test the binding ability of OV71 and D9. The his-OV20ΔC protein served as a negative control that is unable to bind dsRNA [44]. Proteins bound to polyI: C were detected by Western blotting using an anti-His antibody. **I**: input (10% of binding reaction); **E**: eluted fractions from the beads; **B**: beads

**Fig. 5:**
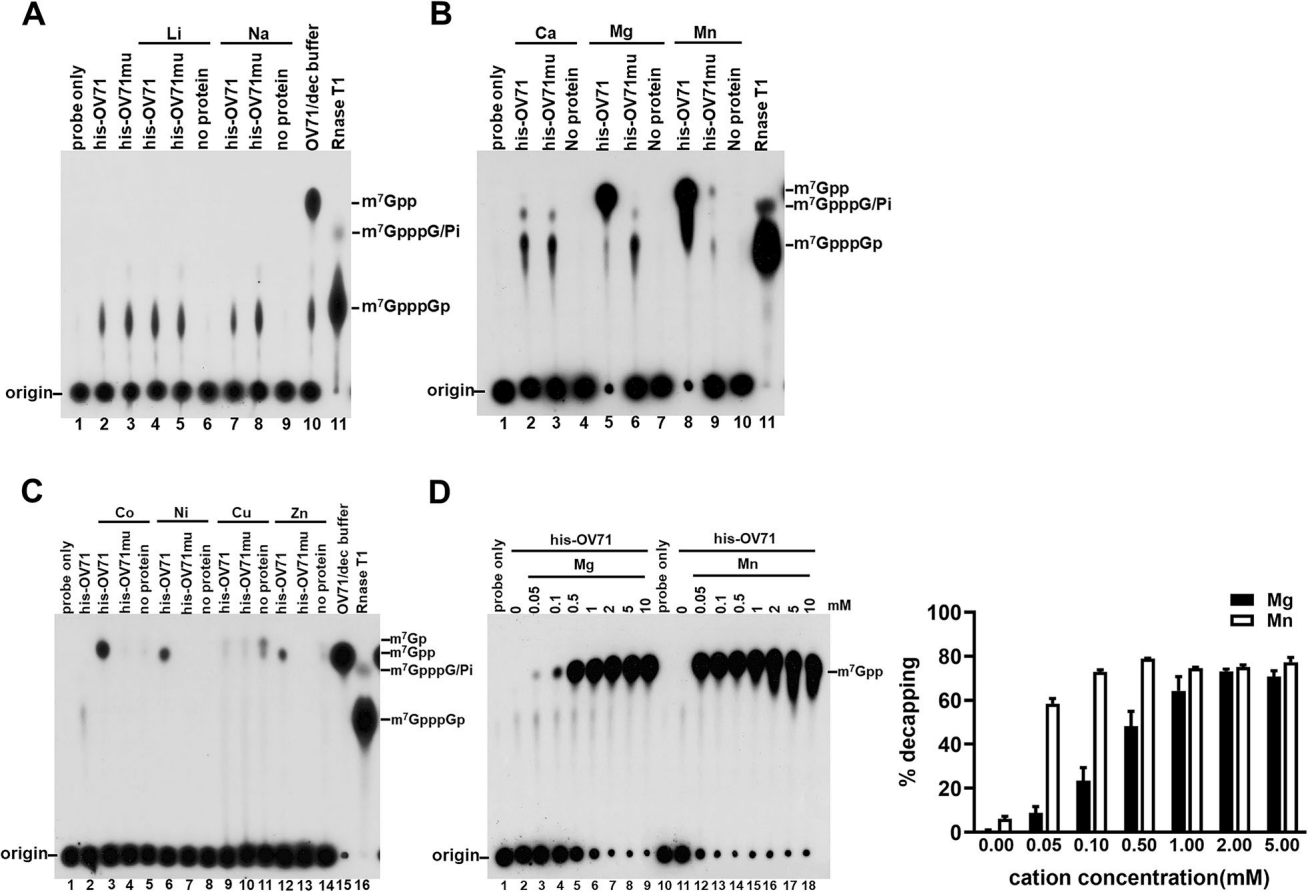
OV71 displays optimal decapping activity in the presence of Mn^2+^. Decapping assays were performed using 4 pmol of his-OV71 or catalytic mutant his-OV71mu (harboring E129Q/E130Q mutations) incubated with radiolabeled capped RNA in a minimal decapping buffer containing 100 mM potassium acetate, 10 mM Tris-HCl, and 2 mM DTT (pH 7.5). Various 2 mM monovalent or divalent metal ions were added as indicated, and reaction products were resolved on TLC plates. (**A**) Reactions with LiCl and NaCl; (**B**) CaCl_2_, MgCl_2_, and MnCl_2_; (**C**) CoCl_2_, NiSO_4_, CuSO_4_, and ZnCl_2_. To determine the identity of the slower migrating intermediates, the capped RNA probe was digested with RNase T1 to generate m^7^GpppGp (lane 11 in (**A**) and (**B**); lane 16 in (**C**)). To determine the position of m^7^GDP, decapping reactions with his-OV71 in the standard decapping buffer (dec buffer) were included (lane 10 in (**A**); lane 15 in (**C**)). Estimated migration positions of m^7^GpppG and inorganic phosphate (Pi) [69] are also indicated. (**D**) Dose-dependent decapping activity of OV71 with increasing concentrations of MgCl_2_ or MnCl_2_. Decapping percentages were quantified as described in Fig. 3E and plotted (right panel of **D**). The error bars represent the standard error of the mean (SEM)

To assess whether recombinant OV71 directly binds single-stranded RNA (ssRNA), we performed electrophoretic mobility shift assay (EMSA) and RNA pulldown assays. His-tagged OV71 and OV71mu (harboring E129Q/E130Q mutations that abolish decapping activity) were purified by cobalt-chelated affinity chromatography, whereas GST (glutathione S-transferase) tagged-OV71 (GST-OV71) and GST-OV71mu fusion proteins were purified using glutathione Sepharose columns. All purified proteins used in the experiments shown in Figs. 4 and 5 were resolved by SDS-PAGE (Fig. 4A). In the EMSA, a 206 nt internally radiolabeled uncapped RNA was in vitro transcribed, gel-purified, and incubated with his-OV71 or his-OV71mu. Bovine serum albumin (BSA) was used as a negative control, as it has been previously used as a negative control in another study [58]. Both his-OV71 and his-OV71mu shifted with the RNA probe (Fig. 4B, lanes 3–4 and 6–7), indicating direct RNA binding and demonstrating that the E129Q/E130Q mutation in the Nudix motif does not impair the RNA-binding ability. To further validate the RNA-binding ability of OV71, we performed pulldown assays using biotinylated ssRNA. GST-OV71, GST-OV71mu, his-OV71 and his-OV71mu were all efficiently pulled down by the RNA (Fig. 4C), confirming RNA-binding ability for both wild-type and mutant OV71 proteins. The amino acid sequence of OV71 contains clusters and dispersed basic residues that may serve as potential RNA-binding sites (Fig. 4D). These positively charged residues were mapped onto the AlphaFold2.0-predicted OV71 structure (Fig. 4E). The predicted surface charge on the same structures, viewed from the same angles as in Fig. 4E, is shown in Fig. 4F. Both Fig. 4E and F show that individually dispersed basic residues can spatially converge to form distinct positively charged surface patches, including those formed by R25 and H30, R82 and R157, as well as R78 and R83. Notably, positively charged residues also surround the Nudix motif (Fig. 4E and F), potentially interacting with the RNA region adjacent to the cap and thereby supporting the interaction between the Nudix motif and the cap structure.

Poxviruses generate double-stranded RNA (dsRNA) from complementary viral RNA strands transcribed from the DNA strands in opposite directions [59]. dsRNA serves as an important pathogen-associated molecular pattern (PAMP) that can be recognized by pattern recognition receptors (PRRs) to induce innate antiviral responses [60]. A previous study reported that D9 and D10 prevent the accumulation of viral dsRNA [33], suggesting that these two proteins may associate with dsRNA and cleave its cap, which facilitates its degradation by nucleases. Another recent report showed recombinant D9 binds dsRNA in vitro [30]. In this study, we investigated whether OV71 can also bind dsRNA. Biotinylated polyI: C, a synthetic dsRNA analog, was used in pulldown assays. The orf virus protein OV20.0 is a known dsRNA-binding protein, and its C-terminal truncation mutant OV20ΔC, which lacks dsRNA-binding ability [44], served as a negative control. Both his-OV71 and his-D9 bound to poly I: C (Fig. 4G), suggesting that these two decapping proteins may interact with viral dsRNA to remove its cap.

### OV71 exhibits optimal decapping ability in the presence of Mn^2+^

Nudix enzymes require divalent cations for their catalytic activity [61], with magnesium (Mg^2+^) and manganese (Mn^2+^) being the most commonly used in reaction buffers. For example, an archaeal ADP-ribose pyrophosphatase exhibits optimal activity with Mg^2+^ [62], while yeast and mammalian Dcp2 enzymes, as well as the vaccinia virus decapping enzyme D10, prefer Mn^2+^ [28, 63, 64]. In addition to Mg^2+^ and Mn^2+^, the human Nudix enzyme Nudt3 has exhibited catalytic activity in the presence of Co^2+^ and Zn^2+^, depending on the substrate specificity [65]. Furthermore, other divalent cations, such as Ca^2+^, Cu^2+^, and Ni^2+^, are also biologically relevant as they are cofactors for various enzymatic activities [66–68]. To investigate the cation preference of OV71, we tested its decapping activity in the presence of a panel of monovalent and divalent cations, including Li^+^, Na^+^, Mg^2+^, Mn^2+^, Ca^2+^, Co^2+^, Ni^2+^, Cu^2+^, and Zn^2+^. The standard decapping buffer used in Figs. 2 and 3 contains 2 mM Mg^2+^ and 0.5 mM Mn^2+^ as described in Materials and Methods. For the experiments shown in Fig. 5, we used a minimal decapping buffer containing K^+^ but lacking divalent cations (100 mM potassium acetate, 10 mM Tris-HCl, and 2 mM DTT, pH 7.5) and then supplemented it with individual metal ions. OV71 showed no decapping activity in the minimal buffer alone (Fig. 5A, lane 2) or in the presence of 2 mM supplemented Li^+^, Na^+^, Ca^2+^, or Cu^2+^ (Fig. 5A, lanes 4 and 7; Fig. 5B, lane 2; Fig. 5C, lane 9). By contrast, robust decapping activity was observed with Mg^2+^ and Mn^2+^ (Fig. 5B, lanes 5 and 8), moderate activity with Co^2+^ and Ni^2+^ (Fig. 5C, lanes 3 and 6), and marginal activity with Zn^2+^ (Fig. 5C, lane 12). Trace levels of m^7^GMP and m^7^GDP detected in Cu^2+^-containing reactions (Fig. 5C, lanes 9–11) also appeared in the no-protein control (lane 11), suggesting that these products may result from non-enzymatic decomposition of the cap structure, rather than the OV71 activity. We further examined the effect of different Mg^2+^ and Mn^2+^ concentrations on OV71 activity. As shown in Fig. 5D, the enzyme exhibited optimal decapping activity with 2 mM Mg^2+^ or 0.5 mM Mn^2+^, consistent with previous findings for human Dcp2 [28] and the vaccinia virus D10 [64]. In the latter report, a high concentration of Mg^2+^ failed to yield optimal decapping activity, likely due to the use of a high substrate concentration: the enzyme-to-substrate molar ratio was 1:0.5 in the Soulière et al.. study [64] as opposed to 1:0.0125 in our study.

### OV71 is expressed early during the viral replication cycle

**Fig. 6:**
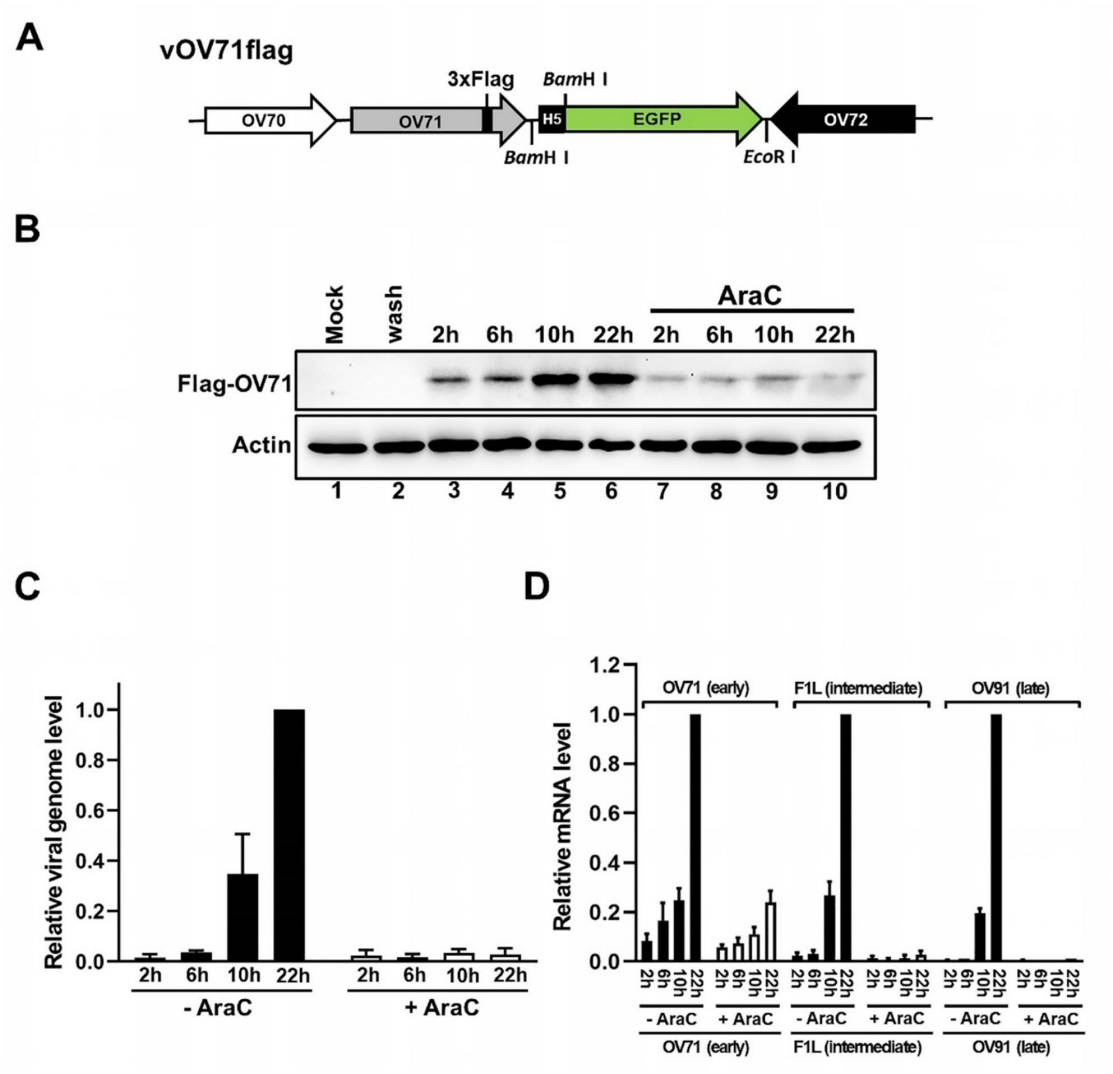
OV71 is expressed early during viral replication. (**A**) Schematic representation of the recombinant orf virus vOV71flag. A 3xFlag tag was inserted at the 3′ end of the OV71 open reading frame. An EGFP gene driven by a modified vaccinia virus H5 promoter, was inserted between OV71 and OV72 open reading frames. Arrows indicate the direction of transcription. (**B**) Temporal expression of Flag-tagged OV71 (Flag-OV71) in the presence or absence of cytosine arabinoside (AraC). Goat fibroblast (FB) cells were infected with vOV71flag at an MOI of 12 and harvested at 2 h, 6 h, 10 h and 22 h postinfection (lanes 3–10). In lane 2, the virus was added and immediately washed away with PBS, followed by three additional washes. Cell lysates were analyzed by Western blotting using anti-Flag and anti-Actin antibodies. (**C**) Relative viral genome levels in vOV71flag-infected FB cells in the presence or absence of AraC. DNA extracted from infected cells was subjected to qPCR using primers targeting the OV71 gene. Relative viral genome levels were determined by normalizing each absolute genome copy number to that of the 22 h sample without the AraC treatment. (**D**) Temporal expression of OV71 (early gene), F1L (intermediate gene), and OV91 (late gene) in vOV71flag-infected FB cells, determined by RT-qPCR. The RNA levels were normalized to those of the 22 h samples without the AraC treatment in each gene group. The error bars in (**C**) and (**D**) represent the standard error of the mean (SEM)

After characterizing the in vitro biochemical properties of recombinant OV71, we next investigated the temporal expression of the OV71 gene during the viral replication cycle. Poxvirus gene expression is temporally programmed into three stages: early, intermediate, and late, defined by their timing after infection [70]. The expression of early genes depends on the virion-packaged factors that become active immediately upon infection. Intermediate gene expression requires early proteins and occurs on newly replicated viral DNA. Late gene expression depends on intermediate proteins and also requires newly replicated viral DNA. Therefore, early gene expression does not require DNA replication, whereas intermediate and late genes are postreplicative genes that are expressed only after DNA replication [71, 72]. Temporal transcriptional profiles of vaccinia virus and orf virus genes have been determined by microarray [73, 74] and RNA-seq [71, 72, 75] approaches. A recent study by Joshi et al. [72]. classified OV71 as an early gene, based on the detection of its mRNA as early as 1 h postinfection in primary ovine cells, before viral DNA replication. To confirm the temporal expression of OV71 at the protein level, we generated a recombinant virus vOV71flag, in which a 3xFlag tag was inserted at the 3′ end of the OV71 open reading frame in the wild-type orf virus genome (Fig. 6A). Goat fibroblast (FB) cells were infected with vOV71flag at an MOI of 12 in the presence or absence of cytosine arabinoside (AraC), an inhibitor of DNA replication. Infected cells were harvested at various time points postinfection for Western blot analysis. Flag-tagged OV71 (Flag-OV71) was detected first at 2 h postinfection, and persisted into later stages of infection (Fig. 6B, lanes 3–6). Given that the cell monolayers were thoroughly washed with PBS following viral adsorption to remove unbound virions and factors, the detected Flag-OV71 signal represented the *de novo* synthesized protein from the replicating virus in infected cells, rather than the unbound input protein from the infecting viral solution. Moreover, no Flag-OV71 signal was detected in lane 2 of Fig. 6B, where the input viral solution was added and then immediately washed off without allowing time for adsorption, indicating that the washing step effectively removed all unbound virus and factors. In the presence of AraC, Flag-OV71 was still detected at all time points (Fig. 6B, lanes 7–10), although the signals were weaker. According to Joshi et al. [72], viral genes whose transcripts are detected before 2 h postinfection are classified as early genes. OV71 is an early gene, as its mRNA was detected at 1 h postinfection [72]. The Western blot results in Fig. 6B further support this classification, showing that OV71 protein was expressed before 2 h postinfection (lane 3). AraC did not block its expression, although it was reduced. To verify that viral DNA replication was inhibited by AraC, relative viral genome levels were determined by qPCR (Fig. 6C). In the absence of AraC, viral DNA replication initiated at approximately 6 h postinfection, consistent with previous findings by Joshi et al. [72]. In the presence of AraC, viral genome levels remained at the lowest level at all the time points, indicating that DNA replication was effectively blocked (Fig. 6C). To test whether AraC blocks the postreplicative intermediate and late genes, we selected the intermediate gene F1L and the late gene OV91, based on the classification by Joshi et al. [72]. Due to the lack of commercially available antibodies against orf virus proteins, we used RT-qPCR to examine the mRNA levels of OV71 (early gene), F1L (intermediate gene), and OV91 (late gene). As shown in Fig. 6D, in the presence of AraC, OV71 expression was reduced but not entirely blocked (consistent with the Western blot results in Fig. 6B). In contrast, the expression of F1L and OV91 was nearly abolished in the presence of AraC (Fig. 6D), consistent with the findings reported by Joshi et al. [72]. The reduced expression of OV71 at the early time points (2 h and 6 h in Fig. 6B and D) may reflect a slight inhibitory effect of AraC that targets unknown pathways other than DNA replication. A similar mild inhibitory effect of AraC on early gene expression was also observed in Table 1 of the study by Joshi et al. [72]. The more pronounced inhibition of OV71 expression at later time points (10 h and 22 h in Fig. 6B and D) may be attributed to the limited availability of newly synthesized DNA templates. Although OV71 expression can still build up from the existing DNA templates without the synthesis of new viral DNA, the overall transcript level remained lower.

### Decapping activity of OV71 significantly contributes to efficient viral replication

**Fig. 7:**
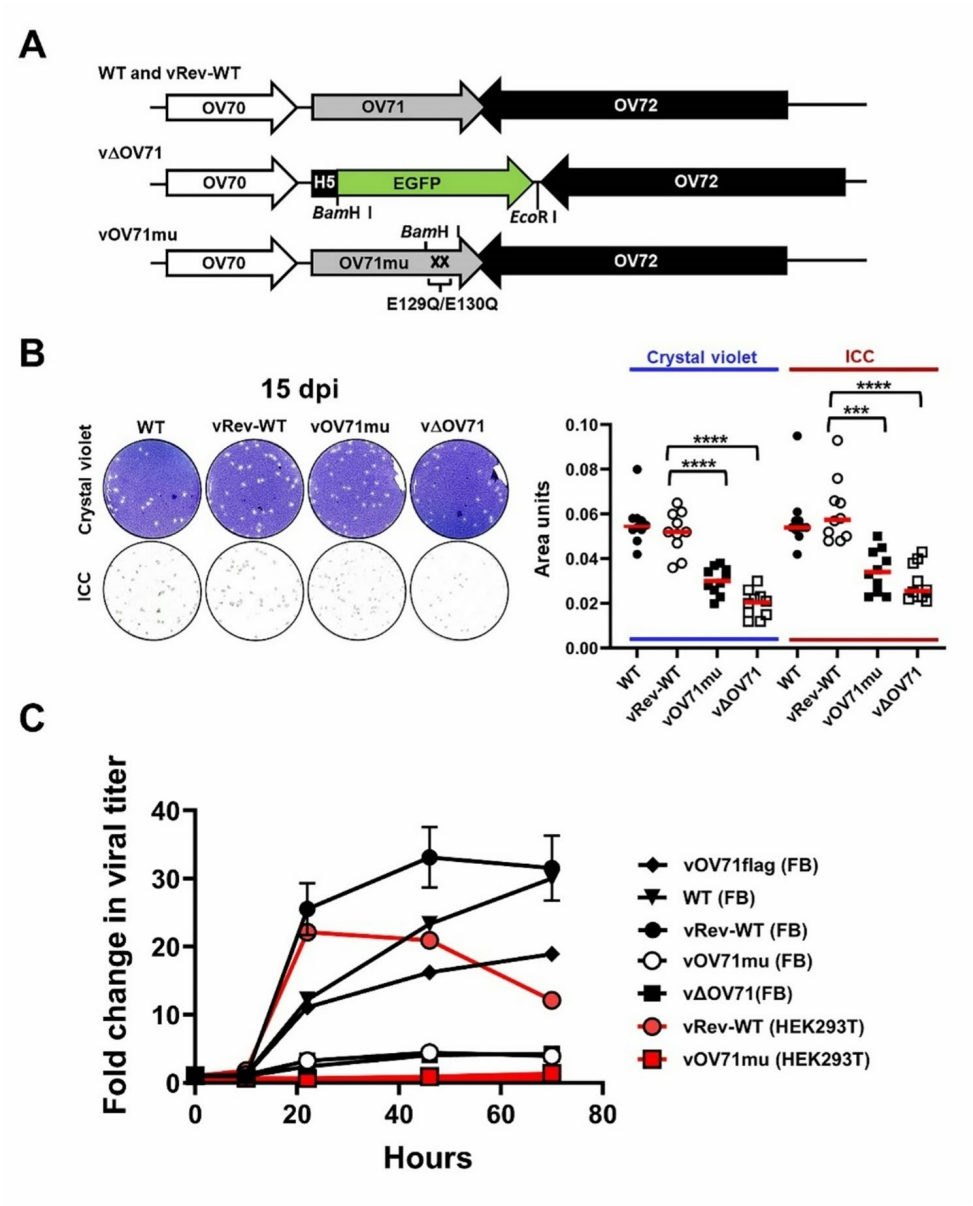
Abrogation of OV71 decapping activity impairs viral replication. (**A**) Schematic diagrams of the genome structures of wild-type (WT) orf virus and the revertant virus (vRev-WT) generated from the OV71 deletion mutant, the OV71 deletion mutant (vΔOV71), and the OV71 catalytic mutant (vOV71mu) in which both E129 and E130 residues were substituted with glutamine (Q). All recombinant viruses were generated by homologous recombination as described in Materials and Methods. (**B**) FB cell lysates infected with WT, vRev-WT, vOV71mu, or vΔOV71 were serially diluted and used to infect fresh FB cells to allow formation of plaques. At 15 days postinfection, cells were stained with 0.1% crystal violet in 75% ethanol or by immunocytochemistry (ICC) using a homemade anti-F1L antibody [48]. Ten well-formed plaques from each well were selected. The area of the cleared (unstained) regions in the crystal violet-stained plaques and the colored regions in the ICC-stained plaques were measured using ImageJ1.53 K (https://imagej.net/ij/) and plotted in the right panel. Statistical significance is denoted by *P* < 0.05 (*), *P* < 0.01 (**), *P* < 0.001 (***), and *P* < 0.0001 (****). (**C**) One-step growth curves of vOV71flag, WT, vRev-WT, vOV71mu, and vΔOV71 in FB cells, and of vRev-WT and vOV71mu in HEK293T cells. Cells were infected at an MOI of 1 and harvested at 0, 10, 22, 46, and 70 h postinfection. Viral titers were determined by infecting fresh FB cells with the diluted infected lysates. Viral titers were normalized to those at the 0 h time point, and the fold change in viral titer is shown on the Y-axis. Data represent three independent experiments for vRev-WT (FB), vΔOV71 (FB) and vOV71mu (FB), and one experiment each for WT (FB), vOV71flag (FB), vRev-WT (HEK293T), and vOV71mu (HEK293T). The error bars represent the standard errors of the mean (SEM)

To investigate the role of OV71 in viral replication, we constructed a deletion mutant virus, vΔOV71, in which the OV71 gene was replaced by an EGFP gene driven by the H5 promoter. We also generated a catalytic site mutant virus, vOV71mu, in which the critical glutamic acid residues (E129 and E130) required for the catalytic activity were substituted with glutamines (Q). A revertant virus, vRev-WT, was created by reintroducing the wild-type OV71 gene into the vΔOV71 genome, replacing the EGFP gene. The maps of these recombinant viruses are shown in Fig. 7A. All viruses were propagated in FB cells, and the infected cell lysates were diluted and used to infect fresh FB cells to allow the formation of plaques. At 15 days postinfection (15 dpi), infected cells were stained with 0.1% crystal violet in 75% ethanol or by immunocytochemistry (ICC) using a homemade anti-F1L antibody [48] that recognizes the orf virus membrane protein F1L. Both staining methods revealed that vΔOV71 and vOV71mu produced smaller plaques than to vRev-WT and wild-type (WT) viruses (Fig. 7B). To evaluate viral replication efficiency, FB or HEK293T cells were infected with WT, vOV71flag, vRev-WT, vOV71mu or vΔOV71 at an MOI of 1, and viral titers were measured at various time points. The strains vOV71flag, WT, and vRev-WT, all of which contain an unaltered Nudix motif within the OV71 open reading frame, showed peak titers that increased by 19–33 fold relative to the 0 h time point in FB cells. Among them, only vRev-WT was tested in HEK293T cells, where it also exhibited a comparable increase in peak viral titer. In contrast, the mutant strains vOV71mu and vΔOV71 exhibited only a 3.9–4.4 fold increase in FB cells, and vOV71mu showed only a 1.35 fold increase in HEK293T cells (Fig. 7C). These results demonstrate that the decapping activity of OV71 significantly contributes to viral replication. Notably, although the Nudix motif is unaltered in the OV71 open reading frame of vOV71flag, this strain did not replicate as efficiently as WT and vRev-WT (approximately 19-fold vs. 30-fold increase; Fig. 7C), indicating that the introduced genetic modifications exerted a moderate negative effect on viral replication. In addition, although vRev-WT in HEK293T cells showed a titer comparable to that in FB cells at 22 h postinfection (22- and 25-fold increases, respectively), its titer declined to only 12-fold increase at 70 h postinfection, compared with a 31-fold increase in FB cells. Together with the markedly lower virus yield of vOV71mu in HEK293T cells, these results indicate that HEK293T cells are less permissive to orf virus replication than FB cells.

### OV71 decapping activity destabilizes host-capped mRNAs while contributing to the accumulation of viral mRNAs

**Fig. 8:**
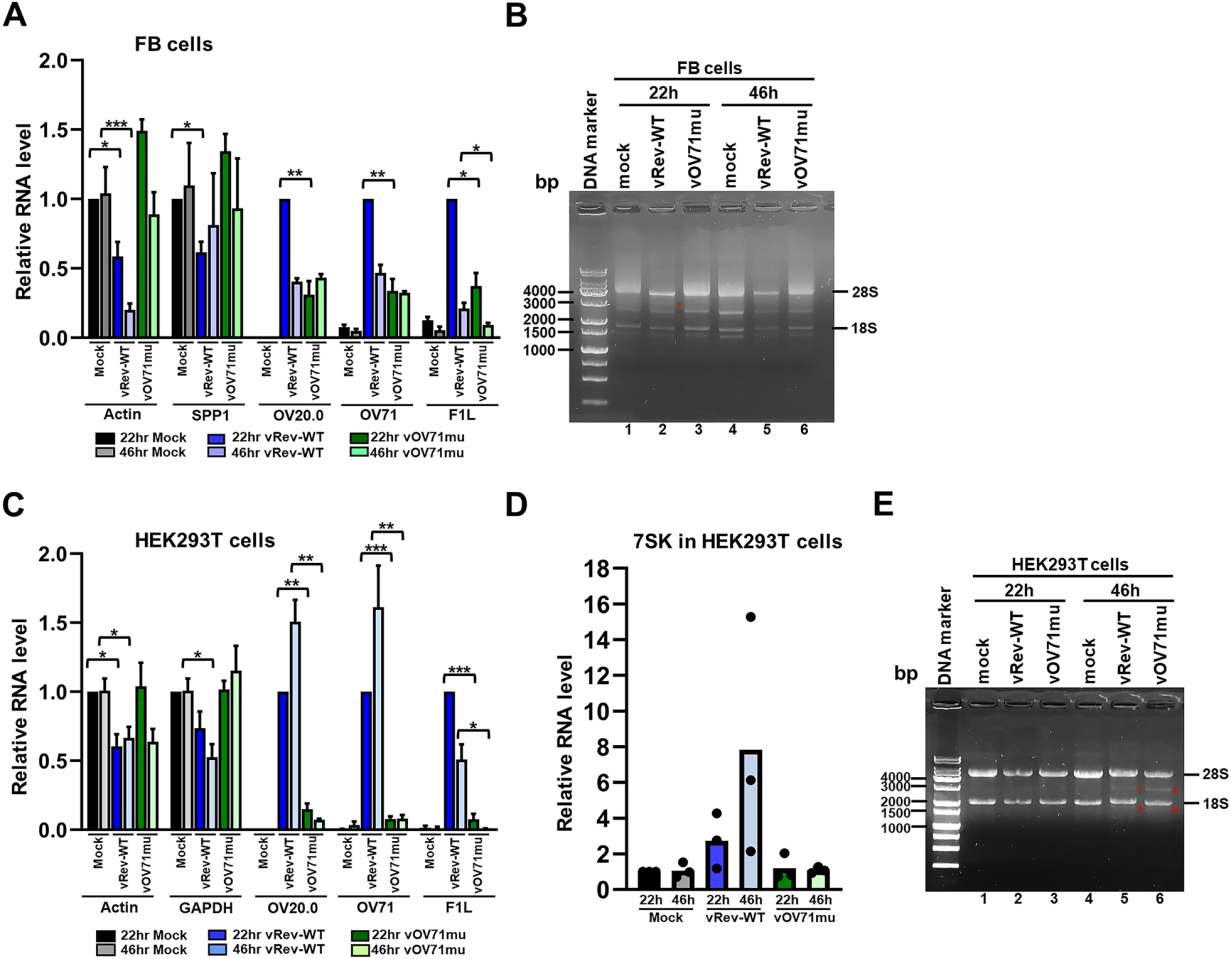
Viral and cellular RNA levels in cells infected with vRev-WT or vOV71mu. Primary goat fibroblast (FB) cells (**A**) and HEK293T cells (**C**) were mock-infected or infected with vRev-WT or vOV71mu at an MOI of 5. Cells were harvested at 22 and 46 h postinfection. Total RNA was extracted and analyzed by reverse transcription-quantitative PCR (RT-qPCR) to quantify cellular β-actin and SPP1 (secreted phosphoprotein 1) mRNAs in FB cells (**A**); β-actin and GAPDH in HEK293T cells (**C**), viral OV20.0, OV71, and F1L mRNAs in both cell types (**A** and **C**); and small nuclear RNA 7SK in HEK293T cells (**D**). Levels of cellular RNAs (β-actin, SPP1, GAPDH, and 7SK) at 22 h and 46 h postinfection were normalized to the mock-infected controls at 22 h postinfection. Viral mRNA levels (OV20.0, OV71, and F1L) at 22 h and 46 h were normalized to each group’s vRev-WT-infected 22 h sample. Data represent three independent experiments performed in triplicate. Statistical significance is denoted as follows: *P* < 0.05(*), *P* < 0.01(**), *P* < 0.001(***), and *P* < 0.0001(****). (**B**,** E**) Total RNA integrity was examined by electrophoresis on glyoxal-denaturing gels. In (**B**), each lane contains RNA (0.5–1.8 µg) extracted from half of the remaining FB cells from a single well of a 12-well plate. In (**E**), each lane contains 1 µg of total RNA extracted from infected HEK293T cells. The positions of 28 S and 18 S rRNAs are marked on the right. Red asterisks indicate rRNA cleavage products. The error bars in (A) and (C) represent the standard errors of the mean (SEM)

Vaccinia virus infection is known to induce widespread degradation of host cell mRNAs [71, 76]. Similarly, recent RNA-seq studies in various cell types have shown that orf virus infection results in downregulation of specific subsets of cellular mRNAs [77–79]. To investigate whether orf virus infection promotes degradation of host or viral mRNAs, and whether this effect is dependent on the decapping activity of OV71, we infected FB and HEK293T cells with either vRev-WT or the catalytic mutant virus vOV71mu at an MOI of 5. Cells were harvested at 22 and 46 h postinfection, and total RNA was extracted for RT-qPCR analysis for selected transcripts, including β-actin and SPP1 (secreted and phosphoprotein 1) for FB cells, and β-actin, GAPDH, and 7SK for HEK293T cells. Notably, 7SK is a small nuclear RNA that contains a noncanonical mpppG cap with a methylated γ-phosphate [80], and is not targeted by the vaccinia virus decapping enzyme D10 in a previous study by Ly et al. [32]. We also examined the expression of viral transcripts OV20.0, OV71, and F1L. As shown in Fig. 8A and C, mRNAs of the selected host genes were markedly reduced in both FB and HEK293T cells infected with vRev-WT compared to the mock-infected cells, while their levels remained stable or were elevated in cells infected with vOV71mu. This result suggests that OV71 mediates the degradation of host-capped mRNAs. As expected, the 7SK mRNA, which possesses a noncanonical cap resistant to hydrolysis by vaccinia virus decapping enzyme D10, did not decrease but instead increased by 3-fold and 8-fold at 22 h and 46 h postinfection, respectively, in cells infected with vRev-WT (Fig. 8D). Interestingly, the levels of viral OV20.0 mRNAs in FB cells infected with vOV71mu were initially lower at 22 h but reached the level comparable to that in vRev-WT-infected cells by 46 h (Fig. 8A), a pattern also observed in cells infected with a D10 deletion mutant of vaccinia virus [24]. However, in HEK293T cells, vOV71mu-infected cells displayed severely reduced viral mRNA levels at both time points (Fig. 8C), consistent with the severely impaired replication of this virus in HEK293T cells (Fig. 7C). Total RNA isolated from infected cells was resolved on denaturing RNA gels (Fig. 8B and E). In Fig. 8B, the total RNA extracted from half of the infected FB cells was loaded into the gel. The rRNA levels were lower in vRev-WT-infected cells compared to those in vOV71mu and mock-infected cells (Fig. 8B, lanes 2 and 5), corresponding to the lower β-actin and SPP1 mRNA levels in Fig. 8A. Since rRNA is not capped and thus not a direct target of OV71, the reduced rRNA may result from a mechanism not directly related to cap hydrolysis. In HEK293T cells, 1 µg of the total RNA extracted from the infected cells was loaded per lane. The rRNA exhibited an equally slight cleavage upon infection with both vRev-WT and vOV71mu at 46 h (Fig. 8E), which did not correlate with the vast difference in viral mRNA levels between the two viruses (Fig. 8C), suggesting it may result from a general antiviral response independent of OV71 decapping activity. The strikingly low levels of viral mRNAs in vOV71mu-infected HEK293T cells may result from impaired transcription of viral genes, or extensive degradation of viral mRNAs by an unidentified mechanism that selectively targets viral mRNAs while sparing host mRNAs and rRNAs. Further experiments are required in the future to explore these possibilities.

## Discussion

In this study, we characterized the biochemical properties of recombinant OV71, the sole Nudix protein encoded by the orf virus genome, and investigated its role in viral replication by comparing the phenotypes of the wild-type revertant and OV71-defective mutant viruses. Amino acid sequence analysis revealed that OV71 shares considerable identity (31.1%) with its vaccinia virus ortholog D10 (Fig. 1A), suggesting potential structural similarity [81]. Indeed, the AlphaFold2.0-predicted structure of OV71 closely resembles those of D10 (also predicted) and D9 (experimentally resolved) (Fig. 1C), despite sharing only 14.6% sequence identity with D9 (Fig. 1A). Overall, the biochemical properties of OV71 are generally consistent with those of known viral Nudix decapping enzymes. Similar to viral decapping enzymes D10, D9, L375, and the human decapping enzyme Dcp2, OV71 hydrolyzes the 5′ cap of mRNA to release m^7^GDP, but is unable to hydrolyze free cap structures, suggesting the requirement of RNA body for the decapping activity (Figs. 2C and D and 3A) [21, 22, 25, 27, 28]. Consistently, uncapped RNA effectively competed for decapping, whereas the cap structure alone did not (Fig. 3B and C), indicating that OV71 has a significantly higher affinity for the RNA body than for the cap itself, suggesting that OV71 may bind the RNA body to position and access the 5′ cap for hydrolysis. The EMSA and ssRNA pulldown assay results confirm that OV71 can directly bind uncapped RNA (Fig. 4B and C). Indeed, both clustered and dispersed basic residues (Fig. 4D) form extended positively charged surface regions surrounding the Nudix motif, which may serve as RNA-binding interfaces (Fig. 4E and F).

Interestingly, OV71 contains a strikingly higher proportion of arginine residues compared to other characterized viral decapping enzymes (Fig. 4D; Table 2). The viral decapping enzymes OV71, D9, D10, g5R and L375 all contain similar proportions of basic amino acids, ranging from 15.3% to 19.4% (Table 2). OV71 stands out by having an exceptionally high fraction of arginine among its basic amino acids, at 72.5%. This percentage is approximately 2–3 times higher than that observed for other enzymes (Table 2). Therefore, OV71 is an arginine-rich RNA-binding protein, despite lacking a recognizable RNA-binding domain as predicted by bioinformatics. Arginine is a unique amino acid with extensive interaction potential with other biomolecules, as its side chain contains a guanidinium group that offers more hydrogen bond donors than the ε-amino group on the lysine side chain. The positively charged guanidinium group also forms salt bridges with negatively charged moieties [82, 83]. Arginine-rich RNA-binding proteins have been identified in various viruses, where they recognize their cognate RNAs often through secondary hairpin structures. Examples include the flock house virus coat protein, which binds the viral RNA genome [84]; the λ phage N protein, which recognizes the hairpin structure in antiterminator RNA [85]; the Tat proteins of human and bovine immunodeficiency viruses (HIV and BIV) [86, 87], and the HIV Rev protein [88]. Arginine has also been reported to effectively stabilize biomolecular condensates, which are membraneless organelles enriched in proteins or nucleic acids. These condensates form through the phase separation of biomolecules, resulting in a dense phase distinct from the surrounding dilute phase [89, 90]. OV71 is likely to form complexes with the hairpin structure in the ssRNA molecules, or with the dsRNA molecules which resemble the stem of the ssRNA hairpin structure, and may localize to membraneless cellular granules similar to stress granules or processing bodies, to carry out specific functions.

**Table 2:**
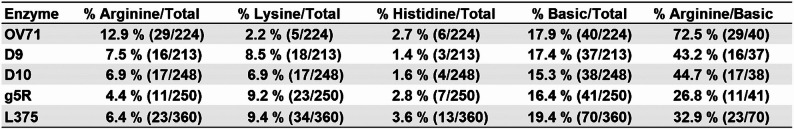
Amino acid composition of viral decapping enzymes

Similar to D10 and hDcp2, OV71 exhibits robust decapping activity with both Mn^2+^ and Mg^2+^, with Mn^2+^ supporting the highest activity (Fig. 5B and D). Specifically, consistent with the findings for hDcp2, OV71 displays peak activity in the presence of 0.5 mM Mn^2+^ and 2 mM Mg^2+^ (Fig. 5D) [28]. Notably, while hDcp2 displays appreciable activity only in the presence of Co^2+^ among divalent cations other than Mn^2+^ and Mg^2+^ [28], OV71 retained considerable activity not only with Co^2+^ but also with Ni^2+^ and Zn^2+^ (Fig. 5C). This broad cation compatibility suggests that OV71 can be readily activated under diverse ionic conditions. Such biochemical flexibility may allow OV71 to remain catalytically active in a broader range of cellular environments.

The role of OV71 in viral replication was investigated using recombinant viruses. The temporal expression of OV71 was assessed using a recombinant virus expressing Flag-tagged OV71 (vOV71flag, Fig. 6A). Expression of Flag-OV71 initiated before 2 h postinfection and was not blocked by AraC (Fig. 6B and D), resembling the expression pattern of ASFV-DP [26]. These results indicate that OV71 is an early protein, consistent with the RNA-seq data reported by Joshi et al. [72]. Notably, the vaccinia virus D9 is also expressed early during infection [91], suggesting that early expression of decapping enzymes is a conserved feature among poxviruses. This temporal pattern suggests that decapping activity plays an important role during the initial stages of infection. Nevertheless, loss of D9 decapping activity does not affect viral replication in cultured BS-C-1 cells [24, 33], likely because the later-expressed D10 compensates for the absence of D9.

Despite this redundancy in viral replication in cultured cells, D9 and D10 exhibited distinct functional characteristics in other contexts. In an animal model, mice infected with the D9 mutant showed a slightly higher virus yield in respiratory tissues compared to those infected with the revertant of the D10 mutant [33]. RNA-seq analysis revealed that while some viral transcripts were targeted by both D9 and D10, distinct subsets were selectively targeted by each enzyme [32]. Retention of the D9-specific transcripts may therefore contribute to the slightly enhanced virulence of the D9 mutant in mice. Together, these observations suggest that differential temporal expression of D9 and D10 targets distinct transcript subsets, thereby allowing more precise regulation of mRNA turnover-mediated pathways.

An intriguing feature of parapoxviruses is that they encode only a single decapping enzyme (similar to African swine fever virus), whereas most other poxviruses encode two. This evolutionary distinction raises key questions about how parapoxviruses regulate mRNA turnover and counteract host immune defenses. Unlike many RNA viruses, which rely on high mutation rates to generate immune-escape variants, double-stranded DNA viruses such as poxviruses exhibit relatively low mutation rates and instead often employ gene amplification to resist host immune pressure [92, 93]. One possibility is that the common ancestor of poxviruses and African swine fever virus originally encoded a single decapping enzyme. As certain descendant lineages expanded their host range and encountered stronger antiviral pressures, duplication of the decapping gene may have occurred and thereby conferring a selective advantage. Over evolutionary timescales, accumulated mutations in the duplicated genes likely drove functional divergence, enhancing viral fitness through more refined control of RNA turnover. These lineages likely evolved into modern poxviruses which encode two decapping enzymes, whereas those retaining a single decapping gene gave rise to parapoxviruses.

Alternatively, parapoxviruses may have emerged later from a two-decapping-gene ancestor and eventually lost one gene during host adaptation. According to Babkin and Shchelkunov [94], parapoxviruses diverged from their co-evolving, two-decapping-gene relatives, the mollucipoxviruses, only about 200,000 years ago, much later than the divergence of other *Chordopoxvirinae* lineages. In this scenario, parapoxviruses and mollucipoxviruses likely share a common ancestor with two decapping genes, and parapoxviruses subsequently lost one gene during host adaptation, as a single decapping enzyme became sufficient for viral replication in their specialized host environment.

During natural infection, the prototype parapoxvirus, orf virus, exhibits a strong tropism for dividing epidermal cells. Compared with other *Chordopoxvirinae* members, orf virus lacks sets of genes for nucleotide metabolism and host response modulation, which may contribute to its restriction to dividing epidermal tissues [95]. The combination of a simplified viral genome and a specialized epidermal environment may have reduced the necessity for the virus to maintain two temporally expressed decapping enzymes to regulate the degradation of RNA and other potential non-RNA substrates.

Although RNA-seq studies in orf virus-infected cells did not reveal global host mRNA degradation at later stages of infection as observed in vaccinia virus-infected cells [71, 77, 79], our RT-qPCR analysis demonstrated that selected host-capped RNAs (β-actin, SPP1, and GAPDH) were targeted by OV71 decapping activity, as their levels were reduced in vRev-WT-infected cells, but not in OV71 mutant-infected cells (Fig. 8A and C). The 7SK small nuclear RNA, which bears a noncanonical mpppG cap [80], was not targeted by OV71 (Fig. 8D). This observation is consistent with a previous RNA-seq study identifying the RNA targets of vaccinia virus D10 [32], further confirming that the reduction of the host capped RNA was due to the decapping action by OV71. Notably, similar to the results reported by Ly et al. [32], the 7SK RNA levels were elevated in the presence of wild-type OV71 due to an unknown mechanism (Fig. 8D).

Although host-capped RNAs remained intact in cells infected with the OV71 mutant, viral mRNA levels were strikingly reduced, particularly in HEK293T cells (Fig. 8A and C). This decrease of viral mRNA correlates with the impaired replication of vOV71mu in both cell types (Fig. 7B and C), suggesting that OV71 plays a crucial role in sustaining viral gene expression during infection. Notably, this pattern differs from the phenotype observed in cells infected with the vaccinia virus D9/10 double mutant [33], where extensive degradation of viral and host mRNAs and rRNAs at late infection stages was attributed to RNaseL activation triggered by the accumulation of viral dsRNA, resulting from impaired decapping [33]. In contrast, OV71 mutant-infected cells exhibited a severe reduction in viral mRNA without apparent host rRNA or mRNA degradation in both cell types (Fig. 8A, C, B and E), suggesting that RNaseL activation was minimal or absent in this context. Moreover, the low levels of viral mRNA in these cells would likely limit the formation of dsRNA, reducing the stimulus for RNaseL activation. In contrast, partial rRNA degradation was observed in vRev-WT-infected FB cells (Fig. 8B), accompanied by a reduction of viral mRNA at the later time point (46 h), supporting the possibility of modest RNaseL activation, possibly induced by the presence of viral dsRNA (despite the presence of OV71 decapping activity). These findings suggest that while RNaseL may contribute to RNA degradation in vRev-WT-infected FB cells, it cannot account for the reduced viral mRNA levels observed in vOV71mu-infected cells, as host mRNAs and rRNAs remained relatively unaffected.

Instead, the severely diminished viral mRNA levels in OV71 mutant-infected cells may result from impaired synthesis of viral mRNAs, rather than enhanced degradation of them. This impairment could arise from dysregulation of various stages of the viral replication cycle, such as viral DNA replication, synthesis of viral transcription factors, or virion assembly. One possibility is that the OV71 hydrolase activity is required to degrade specific host mRNAs or other cellular molecules that would otherwise accumulate and disrupt viral replication stages. In other words, these substrates of OV71 may give rise to host factors that directly or indirectly inhibit key steps in the viral life cycle. As discussed in the Introduction, ASFV g5R hydrolyzes diphosphoinositol polyphosphates (PP-InsPs), which are involved in diverse cellular pathways such as vesicle biogenesis and trafficking, cellular stress responses, and apoptosis, suggesting roles for g5R beyond mRNA degradation [35, 37]. In addition to ASFV g5R, two additional examples of microbial Nudix proteins that hydrolyze non-cap substates highlight the importance of such activities in pathogenicity. The first-identified Nudix enzyme, the *E. coli* antimutator MutT, prevents DNA replication and transcriptional errors by hydrolyzing 8-oxo-dGTP and 8-oxo-rGTP, thereby avoiding the incorporation of these oxidized nucleotides into nascent DNA and RNA [96]. Moreover, plant pathogenic fungi secrete MoNUDIX proteins that degrade PP-InsPs, disrupting phosphate signaling in host plants and promoting virulence [97]. Given that many Nudix proteins exhibit flexible substrate specificity, it is conceivable that OV71 may also target additional non-cap substrates involved in diverse host pathways to facilitate viral replication. Identifying and characterizing these potential substrates will be an important direction for future studies.

The rRNA levels in virus-infected HEK293T cells differ from those observed in FB cells, potentially reflecting a host range effect specific to HEK293T cells. Slight rRNA cleavage was observed in both vRev-WT and vOV71mu-infected HEK293T cells (Fig. 8E), which may be independent of OV71 decapping activity. One possible mechanism is apoptosis, as the orf virus protein ORFV119 has been reported to induce apoptosis [98], and groups of cellular apoptotic genes are upregulated during orf virus infection [78]. To further elucidate the underlying mechanisms of these findings, future efforts will focus on addressing these issues: (1) evaluating the dsRNA levels in cells infected by vRev-WT and vOV71mu; (2) determining whether the dsRNA-mediated antiviral pathways and/or the apoptotic pathways are activated; (3) characterizing differentially expressed genes in cells infected by vRev-WT and vOV71mu by RNA-seq; (4) assessing the integrity of the viral particles of vRev-WT and vOV71mu; (5) examining whether OV71 hydrolyzes PP-InsPs or other non-cap substrates. Together, these efforts will further clarify the functional significance of OV71 in virus-host interactions.

## Conclusions

Our study demonstrates that OV71, the sole Nudix hydrolase encoded by the orf virus, requires engagement with the RNA body to access and hydrolyze the 5′ cap structure of its substrates. OV71 binds both single- and double-stranded RNA, suggesting it may act on both species within infected cells. Biochemical analyses revealed that OV71 preferentially utilizes Mn^2+^ as a cofactor, followed by Mg^2+^, and retains activity with several other biologically relevant divalent cations. This broad cation compatibility likely enables OV71 to remain catalytically active under diverse intracellular conditions. Functional studies show that OV71 targets host-capped mRNAs and promotes their degradation. Moreover, OV71 is critical for efficient viral replication and maintains viral mRNA levels through a mechanism distinct from that of vaccinia virus decapping enzymes.

## Supplementary Information

Below is the link to the electronic supplementary material.

Supplementary Material 1

## Data Availability

The nucleotide sequence of the Orf virus OV71 gene has been deposited in the National Institutes of Health (NIH) genetic sequence database GenBank, under accession number OP820499. Other data and supplementary information are available with the manuscript.

## Abbreviations

APMV: *Mimivirus bradfordmassiliense*
AraC: Cytosine arabinoside
ASFV-DP: African swine fever virus decapping enzyme
BPSV: Bovine papular stomatitis virus
BSA: Bovine serum albumin
DMEM: Dulbecco’s modified eagle medium
EGFP: Enhanced green fluorescent protein
EMSA: Electrophoretic mobility shift assay
FB: Goat fibroblast cells
GST: Glutathione S-transferase
hDcp2: Human Dcp2
HRP: horseradish peroxidase
ICTV: International Committee on Taxonomy of Viruses
IPTG: Isopropyl-β-D-thiogalactopyranoside
MCV: *Molluscipoxvirus molluscum*
MOI: Multiplicity of infection
MYXV: *Leporipoxvirus myxoma*
NDPK: Nucleoside diphosphate kinase
Nudix: **Nu**cleoside **di**phosphates linked to another moiety **X**
ORFV: Orf virus
OV71: ORFV071
PP-InsPs: Diphosphoinositol polyphosphates
PBS: Phosphate buffered saline
PEI: Polyethyleneimine
pLDDT: Predicted local distance difference test
PKR: Protein kinase R
RT-qPCR: Reverse transcription-quantitative PCR
SAM: S-adenosyl methionine
SDS-PAGE: Sodium dodecyl sulphate-polyacrylamide gel electrophoresis
TBE: Tris-borate-EDTA
TLC: Thin-layer chromatography
VACV: *Orthopoxvirus vaccinia*
